# Supplementary material for: Occupational injuries and associated factors among sanitary workers in public hospitals, eastern Ethiopia: A modified Poisson regression model analysis
Source: PLoS One. 2024 Nov 15;19(11):e0310970. doi: 10.1371/journal.pone.0310970 (PMC11567533; doi:10.1371/journal.pone.0310970)
Supplement: S1 File — (PDF) [file pone.0310970.s001.pdf]

Haramaya University College of Health and Medical Sciences **Code:______**

A. English language version For Competent Adults: Ages > 18 Years)

1.Introduction: My name is __________________________________, I am working as a data collector for the study being conducted in this community by Research team (Sina Temesgen Tolera, Tesfaye Gobena, Nega Assefa, Abraham Geremew and Elka Toseva). I kindly request you to lend me your attention to explain you about the study and being selected as the study participant.

2. The study/project title: Burden of occupational Injuries and Determinants among sanitary workers public hospitals in eastern Ethiopia

3. Purpose/aim of the study: The findings of this study can be of a paramount importance for the hospitals and other sectors to plan health and safety practice in the save environment among sanitary workers namely cleaners, waste collectors and others. Moreover, the aim of this study is to write a dissertation as a partial requirement for the fulfillment of a Doctor of Philosophy's Program in Environmental Health for the principal investigator.

4. Procedure and duration: I will be interviewing hospital sanitary workers, namely cleaners, waste collectors and sewage workers using a questionnaire and physical observations to provide me with pertinent data that is helpful for the study. There are 80 questions to answer where I will fill the questionnaire by interviewing them. The interview on each hospital sanitary worker will take about 45-60 minutes.

5. Risks and benefits: The risk of participating in this study is very minimal, but only taking few minutes from sanitary workers' time. There would not be any direct payment for participating in this study. But the findings from this research may reveal important information for the hospital particularly amendment of occupational health and safety guideline, and risk mitigation, which is one part of infection prevention activities in the hospital.

6. Confidentiality: The information that we will be provided will be kept confidential. There will be no information that will identify the participants in particular. The findings of the study will be general for the study community and will not reflect anything particular of individual persons. The questionnaire will be coded to exclude showing names. No reference will be made in oral or written reports that could link participants.

7. Rights: Participation for this study is fully voluntary. The participants have the right to declare to participate or not in this study. If they decide to participate, they have the right to withdraw from the study at any time and this will not label them for any loss of benefits which they otherwise are entitled. They do not have to answer any question that they do not want to answer.

8. Contact address: If there are any questions or enquires any time about the study or the procedures, please contact Principal Investigator: Sina Temesgen: sinatem3@gmail.com; +251913023634; Institutional Health Research Ethics Review Committee (IHRERC) at office phone 0254662011 or P.O.Box 235, Harar, Ethiopia

9. Declaration of informed voluntary consent: I have read/ was read to me the participant information sheet. I have clearly understood the purpose of the research, the procedures, the risks and benefits, issues of confidentiality, the rights of participating and the contact address for any queries. I have been given the opportunity to ask questions for things that may have been unclear. I was informed that I have the right to withdraw from the study at any time or not to answer any question that I do not want. Therefore, I declare my voluntary consent to participate in this study with my initials (signature).

Name and signature of participant: ________________________ ___________ Date _____________

Name and signature of Data Collector: __________________ ___________ Date _____________

N.B

This is signed face to face in the presence of the data collector[If Participants refuse to participate please ask the reason and take note]

Please provide a copy of this signed consent to the participant.

If the participant is a lay person and cannot sign initials, can put his/her thumb print in front of a competent witness; and the witness has to sign alongside (with his/her name and address).

S.n	1. Socio demographic characteristics			
01	Employment: 1 Permanent	2. Contract	3. Outsourced	4.Other
02	Sex	Male	Female	
03	Age:	____________________		
04	Work Experience	____________________		
05	Educational status	____________________		___________
06	Marital Status (Tick√) Single	Married	Separated	Divorced
07	Income monthly salary ______________			
08	Job categories (Tick√): Cleaners	Waste Collectors	Waste emptier	
	Type of your shift[Job rotation]? _____ 1st shift ______2nd shift_____3rd shift_ _ There is no Shift ______			
2. Occupational Injuries :Say "Yes if you are injured and No if not				
09	Had you injured last 12 months due to occupational related exposure?		Yes	No
#	If "YES" #09, what is frequency of the injury? Once	Twice	More than two	
#	If "YES" #09, what was the Type/s of injury? (May you [√] more than one injured body			
	Contusion (includes bruise)<br>General Cut<br>Body Scratch<br>Abrasion/Rough cut/<br>Laceration/Deep cut	Dislocation /<br>Puncture<br>Fracture<br>Allergy and irritation<br>Amputations (body Type _____________)		
#	If "YES" #09, which "Parts of body injured [Tick [√] one or more)			

	Finger Arms Legs feet/toe			
	Head Teeth Eye Other__________			
#	If "YES" #09, what was/were the causes of injury? Tick [√] one or more)			
	1.Sharp or needle injuries<br>2. Falls<br>3. Hand tools<br>4. Slip	5. Hit by falling objects<br>6. Splintering the waste into pieces<br>7. Misuse and ignorance PPE<br>8. Fighting with your coworkers/boss/		
#	If "YES" #09, how much working days lost /absenteeism/ due to injuries?	________Days		
**3.**	**Other occupational related acquired diseases or impairment**			
			Yes	No
10.1	Before you started your work in the Hospital, do have other health problems?<br>If yes! Mention them: ____			
10.2	After you started your work in the hospital, Is there any other health problems faced with?<br>If Yes, Mention them:____			
10.3	Did you develop Musculoskeletal Disorder due to work related work conditions?			
#	If yes for #10.3, how many days you lost your work due to MSDs? Please write here:<br>________ days			
4. Knowledge				
11	Do you know the chance of hepatitis infection due to contaminated waste?			
12	Do you think needle stick injury is one of your occupational risks?			
13	Do you know that the hospital facility is highly infectious			
14	Do you know pieces of sharps and syringes and needles the causes for occupational injuries?			
15	Do you know about occupational health and safety service at your settings			
16	Do you know the precautions of safe disposal for needles and any sharp wastes?			
17	Do you know occupational health hazards could due to unsafe working conditions			
18	Do you know nosocomial infections can be transmitted through blood			
19	Do you think work load and beyond normal capacity could be resulted occupational problems?			
20	Do youS know nosocomial infections can be transmitted through body fluid contamination			

5	Attitude:<br>Where: 1:Strongly, 2:Disagree, 3:Neutral Disagree; 4: Agree; 5:Strongly Agree)	1	2	3	4	5
21	I believe my chances of developing an occupational illness are great					
22	I feel that I have good chance of getting an occupational illness in my career					
23	I know people in this career field who have an occupational illness					
24	I am aware of post exposure prophylaxis for the prevention of biological hazard					
25	I believe that following standard precautions like PPE can decreases the work risk					
26	Training can reduce risk from occupational health and safety					
27	The thought of getting an occupational illness is deeply concerning					
28	If I developed an occupational illness, my career would be in jeopardy					
29	I believe I'm free from occupational health and safety hazards.					
30	I believe the chances of OHS hazards are relatively small or even insignificant					

6. Associated Factors of Occupational Injuries		Yes	No
31	Did you get occupational and health safety training yet?		
32	Do have sleep disorder/disturbance due to work related problems?		
33	Currently, do you consume an alcohol?		
34	Have you currently have work load?		
35	Do you currently work more 8 hr/day?		
36	Do you currently chew khat?		
37	Currently do you smoke tobacco?		
38	Do you have job stress?		
39	How you satisfy with your current job?		
40	How you satisfy with the work environment?		
41	Is there social recognition for your work?		

7	Personal Protective equipment/PPE Utilization, Supply and comfortable (Yes/No)	Yes	No
42	How frequent use of personal protective equipment?		
43	Are Personal Protective equipment comfortable?		
44	How frequent use of personal protective equipment?		
45	Personal protective equipment interferes with my ability to do my job		
46	Personal protective equipment interferes with my ability to do my job		
47	Personal protective equipment is not always available to me		
48	Wearing PPE will prevent me from future the health problems may occurs due to work		
49	PPE prevents exposure to the kinds of hazards I am around on the job		
50	I don't worry about getting an occupational illness when wearing Personal protective equipment		
51	I benefit by wearing Personal protective equipment		
52	A reminder from my supervisor everyday would be important to my wear of Personal protective equipment		
53	My supervisor checking on me would improve my wear of Personal protective equipment		
54	The threat of disciplinary action is an important factor in ensuring I wear Personal protective equipment		
8.	**Infection prevention and control Questions (Yes/No)**		
55	Do you practice infection prevention and control at your hospital?		
56	Do you wear personal protection equipment when going about your regular business?		
57	Do you practice spilling bodily fluids, such as blood, while collecting waste?		
58	Do you wash your hands properly after finished your work?		
59	Do you separate waste at the source?		
60	Do you dispose of medical waste in a safe manner?		
61	Do you practice good cough and respiratory hygiene?		
62	Do you base your work on the practice of feeding people?		
63	Do you keep needles and dangerous objects in the safety box?		
64	Do you dispose of medical waste in yellow containers?		

Thank you very much for you in advance!
