# Supplementary material for: Occupational injuries and associated factors among sanitary workers in public hospitals, eastern Ethiopia: A modified Poisson regression model analysis
Source: PLoS One. 2024 Nov 15;19(11):e0310970. doi: 10.1371/journal.pone.0310970 (PMC11567533; doi:10.1371/journal.pone.0310970)
Supplement: S2 File — (PDF) [file pone.0310970.s002.pdf]

Yuunivarsiitii Haramaya Kolleejjii Fayyaa fi Saayinsii Fayyaa Koodii:______

A. Afaan Oromo language version Ga'eessota gahumsa qabaniif: Umurii > Waggaa 18

1.Sensa :: Maqaan koo ________________________________, qorannoo hawaasa kana keessatti garee Qorannoo (Sina Temesgen Tolera, Tesfaye Gobena, Nega Assefa, Abraham Geremew and Elka Toseva) tiin gaggeeffamaa jiruuf ragaa walitti qabaa ta'ee hojjechaa jira. Waa'ee qorannichaa fi hirmaataa qorannichaa ta'ee filatamuu kee akka siif ibsuuf xiyyeeffannoo keessan akka naaf liqeessitan kabajaan isin gaafadha.

2. **Mata duree qorannichaa/pirojektichaa**: Ba'aa Miidhaa fi Murteessitoota hojii hojjattoota qulqullinaa hospitaalota mootummaa baha Itoophiyaa

3. **Kaayyoo/kaayyoo qorannichaa**: Argannoon qorannoo kanaa hospitaalotaa fi dameewwan biroo hojjettoota qulqullinaa jechuunis qulqulleessitoota, balfa walitti qabuu fi kanneen biroo biratti naannoo qusannaa keessatti hojii fayyaa fi nageenyaa karoorfachuuf barbaachisummaa olaanaa qabaachuu danda'a. Kana malees, kaayyyoon qorannoo kanaa qorataa muummichaaf Sagantaa Doktara Falaasama Fayyaa Naannoo guutuuf akka barbaachisummaa gartokkeetti dissertation barreessuudha.

4. **Hojimaataa fi yeroo**: Hojjettoota qulqullina hospitaalaa, jechuunis qulqulleessitoota, balfa walitti qabuu fi hojjettoota bishaan xuraa'aa gaaffii fi ilaalcha qaamaa fayyadamuun ragaa barbaachisaa qorannichaaf gargaaru naaf kennuuf gaaffii fi deebii nan taasisa. Gaaffilee 80 ta'an deebii kennuudhaaf bakka ani gaaffii fi deebii gochuun guuta. Af-gaaffiin tokkoon tokkoon hojjetaa qulqullina hospitaalaa irratti taasifamu gara daqiiqaa 45-60 fudhata.

5. Balaa fi faayidaa: Balaan qorannoo kana irratti hirmaachuu baay'ee xiqqaadha, garuu yeroo hojjettoota qulqullinaa irraa daqiiqaa muraasa qofa fudhachuudha. Qorannoon kun hirmaachuuf kaffaltiin kallattiin hin jiraatu ture. Garuu argannoon qorannoo kana irraa argame odeeffannoo barbaachisaa hospitaalichaaf keessumaa fooyya'iinsa qajeelfama fayyaa fi nageenya hojii, fi balaa hir'isuu, kunis qaama hojii ittisa infekshinii hospitaalichaa keessaa tokko ta'e mul'isuu danda'a.

6. Iccitii: Odeeffannoon nuuf kennamu iccitii ta'ee ni eegama. Odeeffannoon addatti hirmaattota adda baasu hin jiraatu. Argannoon qorannichaa hawaasa qorannichaaf waliigalaa kan ta'u yoo ta'u, waan addaa namoota dhuunfaa kan hin calaqqisiifne ta'a. Gaaffiin maqaa agarsiisu akka hin dabalamneef koodii ni kennama. Gabaasa afaaniin ykn barreeffamaan hirmaattoota walitti hidhuu danda'u keessatti eeruun hin kennamu.

7. Mirga: Qo'annoo kanaaf hirmaannaan guutummaatti fedhii ofiitiin kan kennamudha. Hirmaattonni qorannoo kana irratti hirmaachuu fi dhiisuu isaanii labsuuf mirga qabu. Yoo hirmaachuuf murteessan yeroo barbaadanitti qorannicha keessaa ba'uuf mirga kan qaban yoo ta'u kunis faayidaa kasaaraa karaa biraatiin isaaniif malu kamiifuu isaaniif hin mallatteessu. Gaaffii deebii kennuu hin barbaanne kamiifuu deebisuu hin qaban). I kindly request you to lend me your attention to explain you about the study and being selected as the study participant.

8. Teessoo quunnamtii: Waa'ee qorannichaa ykn hojimaata yeroo kamiyyuu gaaffiin ykn gaaffiin yoo jiraate, maaloo Qorataa Muummee: Sina Temesgen: sinatem3@gmail.com qunnamaa; +251913023634 irratti kan argamu; Koreen gamaaggama naamusa qorannoo fayyaa dhaabbilee (IHRERC) waajjira bilbila 0254662011 ykn P.O.Box 235, Harar, Ethiopia

9. Ibsa hayyama tola ooltummaa beekumsa qabu: Waraqaa odeeffannoo hirmaataa dubbiseera/ naaf dubbifameera. Kaayyoo qorannichaa, hojimaata, balaa fi faayidaa, dhimmoota iccitii, mirga hirmaachuu fi teessoo quunnamtii gaaffii kamiifuu sirriitti hubadheera. Wantoota ifa hin taane ta'uu danda'aniif gaaffii akkan gaafadhu carraan naaf kennameera. Yeroo barbaadetti qo'annoo keessaa ba'uuf ykn gaaffii ani hin barbaanne kamiyyuu deebisuuf mirga akkan qabu naaf himameera. Kanaafuu, qorannoo kana irratti hirmaachuuf fedhii kootiin hayyama koo qubee jalqabaa (mallattoo) kootiin nan ibsa.

Maqaa fi mallattoo hirmaataa: _______________________ __________ Guyyaa ____________ .

Maqaa fi mallattoo Walitti qabaa Odeeffannoo: _________________ __________ Guyyaa ____________ .

Hub

Kunis bakka walitti qabaan daataa jirutti fuula fuulatti mallatteeffameera[Yoo Hirmaattonni hirmaachuu didan sababa isaa gaafadhaa hubadhaa].

Maaloo waraabbii hayyama mallattaa'e kanaa hirmaataaf kenni.

Hirmaataan nama laayyoo ta'ee fi qubee jalqabaa mallatteessuu kan hin dandeenye yoo ta'e, ragaa gahumsa qabu fuulduratti qubbee harkaa isaa kaa'uu kan danda'u yoo ta'e; akkasumas ragaan cinatti (maqaa fi teessoo isaa waliin) mallatteessuu qab

Lak	1. Amaloota hawaas-dimoogiraafii		
01	Haala Qaxarrii: 1 Dhaabbata 2. Kontraata 3. Kan liqqeeffatama 4.Kan biroo		
02	Saala	Dhiira	Dhalaa
03	Umurii: ________________________		
04	Muuxannoo hojii _______________________		
05	Gulantaa barumsaa ________________________	___________	
06	Haala gaa'elaa (Kana √ fayyadami) 1.Qofaa 2.Kan fuudhe 3.Kan adda bahe/baate 4.Kan hike/te		
07	Miindaa Ji'aa _________________		
08	Gosa hojii (Tick√): 1. Qulqulleessituu 2. Balfa sassaabduu 3. Balfa Gattuu		
	Dabaree/shiiftii dalagaa/_1. 1ffaa _______ 2. 2ffaaa_____3. 3ffaaa _ _ 4. Shiiftii hin qabu		
**2. Miidhama qaamaa/Balaa sababa saaxilamummaa hojiin walqabatee**			
09	Ji'oota 12 darban balaa sababa saaxilamummaa hojiin walqabatee miidhamteettaa?	Eeyyee	Lakki
#	Yesoo meeqaaf miidhamtee? 1. Yeroo tokko 2. Yeroo lamaaf 3. Lamaa oli		
#	Gosti miidhama kee kanneen armaan gadii keessaa kami?<br>1. Cittoo (dhiita'uu dabalatee) .<br>2. Murannoo Waliigalaa<br>3. Qaama Qaama Barruu<br>4. Cita irra keessaa<br>5. Cita qaama keess seene<br>6. Buqa'uu .<br>7. Qaama uratame<br>8. Caccabuu<br>9. Alarjii fi aarii, (gogaa, ija, sirna hargansuu) .<br>10. Irraa cite bu'uu (Gosa qaamaa)		
#	Yoo #09, "EEYYEE" jette , qaama kee kamtu miidhamee? (tokko ol deebii kennuu dandessu)<br>1. Quba 2. Harka 3. Miila Gulubi/faana<br>5. Mataa 6. Ilkaan 7. Ija Kan biraa yoo jiraate ibsi		
#	Yoo #16, "EEYYEE" jette, sababootni balaa kanaa maalii? (tokko ol deebii kennuu dandessu)<br>1.Wanta qara ykn Lilmootu na waraane<br>2.Kufuudhaanii<br>3.Meesha harkaatti qabadheetu narrti cufe<br>4.Muchuchaachuudhaani<br>5.Wantotatu narratti kufe<br>6. Meeshaa caccabaa balfaa keessatu nawaarne<br>7.Meeshaaleen Eegumsa kenname seeraan itti fayyadamu dhabuuni<br>8. Hojjetaa ykn hogganaa koo waliin walloleeni		
#	Yoo #09, "EEYYEE" jette, yeroo meeqaaf hojii/dalagarraa halftee?	________Guyyoota	
10	Dhukkuboota dalaga waliin wal-qabatanii uumamuu danda'an ykn hanqina		
		Eeyyee	Lakki
10.1	Hojii keessan Hospitaala keessatti osoo hin jalqabin dura rakkoo fayyaa biroo qabduu?<br>Yoo eeyyee ta'e! Asiratti kan caqafame: _______________________________________		
10.2	After you started your work in the hospital, Is there any other health problems faced with?<br>If Yes, Mention them:____		
10.3	Ji'oota darbaaniif Rakkoo Maashaalee Lafee Hojiin walqabatu si mudateeraa		
#	Yoo #10.3 "EEYYEE" jette, yeroo meeqaaf hojii/dalagarraa halftee?		
**3. Rakkoo Maashaalee Lafee wal qabatee sababa dalagaatiin**			
4. Knowledge			
11	Carraa dhukkuba tiruu sababa balfa faalameen qabamuu beektaa?	Eeyyee	Lakki
12	Miidhaan ulee cirrachaa balaa hojii keessanii keessaa tokko jettanii yaaddu?		

13	Dhaabbanni hospitaalichaa baayyee daddarbaa akka qabu beektu		
14	Huccuu fi sibiilli, siriinjii fi cirrachoonni itti fayyadaman, gaazii fi suufii xuraa'e dhukkuba hojiif sababa ta'uu beektu?		
15	Waa'ee tajaajila fayyaa fi nageenya hojii bakka jirtanitti ni beektu		
16	Of eegganoowwan siriinjii fi balfa qara qabu kamiyyuu haala gaariin gatuu ni beektu?		
17	Do know balaa fayyaa hojii sababa haala hojii nageenya hin qabneen dhufuu danda'a		
18	Balaan nageenya hojii sababa itti fayyadama Meeshaaleen Eegumsa Dhuunfaa sirrii hin taane irraa kan ka'e ta'uu danda'a jettanii yaaddu		
19	Infeekshiniin mana yaalaa (nosocomial infections) karaa faalama dhiigaa fi dhangala'aa qaamaa osoo hojjettuu daddarbuu akka danda'u beektu?		
20	Rakkoo maashaalee lafee sababa kee fi haala hojii beekuu		

4.	Hubannoowwaan: 1:Cimsee hin galu, 2:Wali hin galle, 3:Giddu galeessa Walii hin galu; 4: Walii galuu; 5:Cimsee Walii Gala)	1	2	3	4	5
21	Carraan dhukkuba hojiitiin qabamuu koo guddaadha jedheen aman					
22	Dhukkuba hojiin qabamuun na yaaddessa					
23	Hojii koo keessatti sababa dhukkuba hojiitiin saaxilamuun akka danda'u natti dhagahama					
24	Namoota damee hojii kana keessa jiran kanneen dhukkuba hojii qaban nan beeka					
25	Ittisa balaa baayoloojii uumamuuf ittisa saaxilamummaa boodaa (post exposure prophylaxis) ni beeka.					
26	Of eegganoowwan sadarkaa qaban kan akka PPE hordofuun balaa hojii hir'isuu danda'a jedheen amana					
27	Leenjiin balaa fayyaa fi nageenya hojii irraa dhufu hir'isuu danda'a					
28	Yaadni dhukkuba hojiitiin qabamuu baayyee nama yaaddessa					
29	Yoon dhukkuba hojiin qabame hojiin koo balaadhaaf saaxilama					
30	Rakkoon dhukkuba hojii irraa na mudatu umurii koo guutuu naaf tura ture					

**5. Associated Factors of Occupational Injuries**		**Yes**	**No**
31	Leenjii nageenya hojii fi fayyaa argattaniittuu?		
32	Rakkoo irriba jeeqamuu qabduu?		
33	Alkoolii baay'ee dhugduu		
34	Hojii idlee male dalagaa keessaa ol dalagduu?		
35	Guyyaatti sa'aa 8 ol hospitaala keessaa dalagduu?		
36	Jimaa/Chaatii ni qamtuu?		
37	Tamboo ni xuuxxuu/ni dhugduu?		
38	Dhiphina hojii qabdaa?		
39	Hojii kee amma hojjettutti ni gammaddaa?		
40	Naannoo/waajjira hojii ati hojjettutti Gammaddaa		
41	Hojii keessaniif beekamtiin hawaasummaa hin jiraa		

6	**Meeshaalee ittisa dhuunfaa /PPE fayyadamsa, dhiyeessii fi mijetaa (Yes/No)**	**Yes**	**No**
42	Meeshaalee ittisa dhuunfaa yeroo meeqa fayyadamuu?		
43	Meeshaaleen Eegumsa Dhuunfaa mijataadhaa?		
44	Meeshaalee ittisa dhuunfaa yeroo meeqa fayyadamuu?		
45	Meeshaaleen ittisa dhuunfaa dandeettii hojii koo hojjechuu koo gufachiisu		
46	Meeshaaleen ittisa dhuunfaa dandeettii hojii koo hojjechuu koo gufachiisu		
47	Meeshaaleen ittisa dhuunfaa yeroo hunda naaf hin argaman		
48	Meeshaalee ittisa dhuunfaa uffachuun gara fuulduraatti rakkoon fayyaa sababa hojiitiin uumamuu malu akkan hin uumamne na dhorka		
49	Meeshaaleen ittisa dhuunfaa gosoota balaa hojii irratti naannoo koo jiruuf akkan hin saaxilamne taasisa		
50	Meeshaalee ittisa Dhuunfaa yeroon uffadhu dhukkuba hojiitiin qabamuuf hin yaadda'u		
51	Meeshaalee ittisa Dhuunfaa uffachuun fayyadama		
52	Guyyaa guyyaan yaadachiisa supparvaayizara koo irraa dhufu meeshaalee ittisa Dhuunfaa uffachuu kootiif barbaachisaa ta'a		

53	Suppervaayizarri koo na sakatta'uun meeshaalee ittisa Dhuunfaa uffachuu koo ni fooyyessa ture		
54	Balaan tarkaanfii naamusaa Meeshaalee ittisa Dhuunfaa akkan uffadhu mirkaneessuu keessatti waan barbaachisaa dha		
**7.**	**Gaaffilee ittisaa fi to'annoo infekshinii (Eeyyee/Lakki) .**		
55	Hospitaala keessan keessatti ittisaa fi to'annoo infekshinii ni shaakaltuu?		
56	Yeroo hojii idilee keessan deemtan meeshaalee eegumsa dhuunfaa ni uffattaa?		
57	Balfa walitti qabaa osoo jirtuu dhangala'oo qaamaa kan akka dhiigaa dhangalaasuu shaakaltuu?		
58	Hojii keessan erga xumurtanii booda harka keessan sirnaan dhiqattu?		
59	Balfa madda isaa irratti addaan baaftuu?		
60	Balfa yaalaa haala nageenya qabuun ni gattaa?		
61	Qufaa gaarii fi qulqullina sirna hargansuu ni shaakaltuu?		
62	Hojii kee gocha namoota nyaachisuu irratti hundaa'aa?		
63	Saardii fi wantoota balaafamaa saanduqa nageenyaa keessa ni kaa'aa?		
64	Balfa yaalaa meeshaalee keelloo keessatti gattaa?		

Dursinee isiniif guddaa galatoomaa!
