# Supplementary material for: Occupational injuries and associated factors among sanitary workers in public hospitals, eastern Ethiopia: A modified Poisson regression model analysis
Source: PLoS One. 2024 Nov 15;19(11):e0310970. doi: 10.1371/journal.pone.0310970 (PMC11567533; doi:10.1371/journal.pone.0310970)
Supplement: S3 File — (PDF) [file pone.0310970.s003.pdf]

አሳታፊ መረጃ ሉህ እና በፈቃደኝነት ላይ የተመሰረተ ስምምነት ቅጽ

(ብቁ ለሆኑ አዋቂዎች፡ ዕድሜ > 18 ዓመት ኮድ: _______

1. **መግቢያ:** ስሜ ____ እባላለሁ በዚህ ማህበረሰብ ውስጥ በምርምር ቡድን (ሲና ተመስገን ቶሌራ፣ ተስፋዬ ጎበና፣ ነጋ አሰፋ፣ አብርሃም ገረመው እና ኤልካ ጦሴቫ) እየተካሄደ ላለው ጥናት መረጃ ሰብሳቢ ሆኜ እየሰራሁ ነው። ስለ ጥናቱ እና የጥናት ተካፋይ ሆኖ መመረጥዎን ለማስረዳት ትኩረትዎን እንዲሰጡኝ በአክብሮት እጠይቃለሁ።
2. **የጥናቱ/የፕሮጀክቱ ርዕስ፡-** በምስራቅ ኢትዮጵያ በሚገኙ የንፅህና ሰራተኞች የመንግስት ሆስፒታሎች ላይ የሚደርስ የስራ ጉዳት እና ውሳኔዎች ሸክም
3. **የጥናቱ ዓላማ፡-** የዚህ ጥናት ግኝቶች ለሆስፒታሉ እና ለሌሎች ሴክተሮች የጤና እና የደህንነት ስራዎችን በንፅህና አጠባበቅ ሰራተኞች ማለትም በጽዳት ሰራተኞች, በቆሻሻ አሰባሳቢዎች እና በሌሎች ላይ ለማቀድ ከፍተኛ ጠቀሜታ ሊኖረው ይችላል. ከዚህም በላይ የዚህ ጥናት ዓላማ ለዋና ተመራማሪው በአካባቢ ጤና ውስጥ የፍልስፍና ዶክተር ፕሮግራምን ለማሟላት እንደ ከፊል መስፈርቶች የመመረቂያ ጽሑፎችን መጻፍ ነው።
4. **ሂደት እና የቆይታ ጊዜ፡-**የሆስፒታል ንፅህና ሰራተኞችን ማለትም የፅዳት ሰራተኞችን፣ ቆሻሻ ሰብሳቢዎችን እና የፍሳሽ ሰራተኞችን መጠይቅ እና የአካል ምልከታዎችን በመጠቀም ለጥናቱ አጋዥ የሆነ መረጃ እንዲሰጡኝ ቃለ መጠይቅ አደርጋለሁ። መጠይቁን በቃለ መጠይቅ የምሞላባቸው **96** ጥያቄዎች አሉ። በእያንዳንዱ የሆስፒታል የንፅህና ሰራተኛ ላይ የሚደረገው ቃለ መጠይቅ ከ45-50 ደቂቃዎች ይወስዳል፡፡
5. **ጉዳት እና ጥቅሞች:**በዚህ ጥናት ውስጥ የመሳተፍ አደጋ በጣም ትንሽ ነው, ነገር ግን ከንፅህና ሰራተኞች ጊዜ ጥቂት ደቂቃዎችን ብቻ ይወስዳል. በዚህ ጥናት ውስጥ ለመሳተፍ ምንም አይነት ቀጥተኛ ክፍያ አይኖርም። ነገር ግን የዚህ ጥናት ግኝቶች ለሆስፒታሉ ጠቃሚ መረጃን ሊያሳዩ ይችላሉ, በተለይም የሙያ ጤና እና ደህንነት መመሪያ ማሻሻያ, ይህም በሆስፒታል ውስጥ የኢንፌክሽን መከላከል እና ቁጥጥር ተግባራት አንዱ አካል ነው.
6. **ሚስጥራዊነት፡-**የምናቀርበው መረጃ በሚስጥር ይጠበቃል። በተለይ ተሳታፊዎችን የሚለይ ምንም መረጃ አይኖርም. የጥናቱ ግኝቶች ለጥናት ማህበረሰብ አጠቃላይ ይሆናል እናም የግለሰቦችን የተለየ ነገር አያንፀባርቅም። መጠየቂያው ስሞችን ከማሳየት እንዲገለሉ ኮድ ይደረጋል። ተሳታፊዎችን ከጥናቱ ጋር ሊያገናኙ የሚችሉ የቃል ወይም የጽሁፍ ዘገባዎች ማጣቀሻ አይደረግም።
7. **መብቶች፡-** የዚህ ጥናት ተሳትፎ ሙሉ በሙሉ በፈቃደኝነት ነው. ተሳታፊዎች በዚህ ጥናት ውስጥ ለመሳተፍ ወይም ላለመሳተፍ የመግለጽ መብት አላቸው. ለመሳተፍ ከወሰኑ በማንኛውም ጊዜ ከጥናቱ የመውጣት መብት አላቸው እና ይህ ካልሆነ ግን መብት ላላቸው ጥቅማጥቅሞች ኪሳራ አይገልጽም። ሊመልሱት የማይፈልጉትን ጥያቄ መመለስ አያስፈልጋቸውም።
8. **የመገኛ አድራሻ፡-** ስለ ጥናቱ ወይም አሠራሩ ማንኛውም አይነት ጥያቄ ወይም ጊዜ የሚጠይቅ ከሆነ፡ ሲና ተመስገን፡ sinatem3@gmail.com; +251913023634; የተቋማዊ ጤና ጥናትና ስነምግባር ገምጋሚ ኮሚቴ ቢሮ በስልክ ቁጥር 0254662011 ወይም P.O.Box 235, ሀረር, ኢትዮጵያ
9. **በመረጃ ላይ የተመሰረተ የፈቃደኝነት ስምምነት መግለጫ፡-**የተሳታፊውን የመረጃ ወረቀት አንብቤአለሁ/ አንብቤያለሁ። የጥናቱ ዓላማ፣ አካሄዶች፣ ስጋቶች እና ጥቅሞች፣ ሚስጥራዊ ጉዳዮች፣ የመሳተፍ መብቶች እና ለማንኛውም መጠይቆች የእውቂያ አድራሻውን በግልፅ ተረድቻለሁ። ግልጽ ባልሆኑ ጉዳዮች ላይ ጥያቄዎችን እንድጠይቅ እድል ተሰጥቶኛል። በማንኛውም ጊዜ ከጥናቱ የመውጣት ወይም የማልፈልገውን ማንኛውንም ጥያቄ ላለመመለስ መብት እንዳለኝ ተነገረኝ። ስለዚህ፣ በዚህ ጥናት የመጀመሪያ ሆኜ (ፊርማ) ላይ ለመሳተፍ የፈቃዴ ፈቃዴን አውጃለሁ።

- የተሳታፊው ስም እና ፊርማ፡- ________________________________ ____________ ቀን _______________
- የውሂብ ሰብሳቢው ስም እና ፊርማ፡- ___________________________ ____________ ቀን _______________

ማሳሰቢያ

- ይህ መረጃ ሰብሳቢው ባሉበት ፊት ለፊት ተፈርሟል።
- እባክዎ የዚህን የተፈረመ ስምምነት ቅጂ ለተሳታፊው ያቅርቡ።
- ተሳታፊው ተራ ሰው ከሆነ እና የመጀመሪያ ፊደሎችን መፈረም የማይችል ከሆነ የአውራ ጣት አሻራውን ብቃት ባለው ምስክር ፊት ማድረግ ይችላል። እና ምስክሩ አብሮ መፈረም አለበት (ስሙን እና አድራሻውን የያዘ)።

ተ.ቁ	1. የግል ሁኔታ (የተሳታፊ መላያ ኮድ:___________)			
1	**የቅጥር አይነት፡ ቋሚ _____ውል______ ከውጭ የተገኘ ____ሌላ**			
2	ጾታ	**ወንድ**	**ሴት**	
3	እድሜ		____________	
4	የስራ ልምድ		___________	
5	የትምህርት ሁኔታ		____________	
6	የጋቢቻ ሁኔታ ያላገባ/ች ያገባ/ች የተለየ/ች የፈታ/ች			
7	ወራዊ ደመወዝ ____________			
8	የሥራ አይነት 1. ጽዳት 2. ቆሻሻ አሰባሳቢ 3. ቆሻሻ አስወጋጅ			
	የእርስዎ ፈረቃ አይነት: የትኛው ሽፍት ኖት;1ኛ ሽፍት ___ 2ኛ ሽፍት 3ኛ ሽፍት			
**2.ከስራ ጤንነትና ደህንነት ጋር የተያያዙ ጉዳቶች**				
9	ባለፉት 12 ወራት ከስራ ጋር በተገናኘ ጉዳት ደርሶብሃል?	አዎ	የለም	
	ለ#09፣ "አዎ" ከሆነ፤ በአንድ አመት ውስጥ የስራ ላይ ጉዳት ድግግሞሽ ምን ያህል ነበር? ሀ) አንድ ጊዜ ለ) ሁለት ጊዜ ሐ) ከሁለት በላይ			
	ለ#09፣ "አዎ" ከሆነ፤የጉዳት ዓይነቶች ምንድናቸው? (ምልክት ያድርጉ[√] አንድ ወይም ከዚያ በላይ ይቻላል)			
	1. መነሳቀል (ቁስልን ይጨምራል)<br>2. አጠቃላይ መቁረጥ<br>3. የሰውነት መቧጨር<br>4. መበሳጨት / ሻካራ መቁረጥ /<br>5. ሌዘር / ጥልቅ መቁረጥ	6. መፈናቀል /<br>7. መቅጣት<br>8. ስብራት<br>9. አለርጂ እና ብስጭት, (ቆዳ, አይን,የመተንፈሻ አካላት)<br>10. መቆረጥ (የሰውነት ዓይነት		
	ለ#09፣ "አዎ" ከሆነ፤ የትኛው አካልህ/ሽ ላይ የጉዳት ደረሰ? (ምልክት [√] ከአንድ በላይ ይቻላል)<br><br>ሀ) ጣት ለ) ክንዶች ሐ) እግሮች መ) እግር / ጣት<br><br>ሠ) ጭንቅላት ረ) ጥርሶች ሰ) አይን ሸ) ሌላ ካለ ________________			
	ለ#09፣ "አዎ" ከሆነ፤ምክኒያቱ ምንድነው**?** ይህንን ምልክት ይተቀሙ[√] **(ከ1 በላይ መልስ ይቻላል)**			
	1. በስሌት ወይም መርፌ በመወጋት	5. እቃዎች በላዬ ላይ ወድቀው ስለመተኝ		
	2.በመውደቅ	6. ቆሻሻ ውስጥ በሚገኙ ሰባሪ በሆኑ እቃዎች		
	3.በእጆቼ የያዝኩት እቃ ወድቀቆብኝ	7.የተሰጠን የአደጋ መከላከያ በአግባቡ አለመጠቀም		
	4. በሸራተት	8.ከሠረተኛ ወይመ አላቃዪ ጋር በመጣላት		
	ለ#09፣ "አዎ" ከሆነ በጉዳቱ ምክንያት ስንት ቀን ከስራዎት ቀሩ?		________	
**3**	***ሥራ ጋር የተያያዙ በሽታዎች***			
10	ከሥራ ጋር የተያያዙ በሽታዎች ወይም እክሎች		አዎ	የለም
10.1	በሆስፒታል ውስጥ ስራዎን ከመጀመርዎ በፊት, ሌሎች የጤና ችግሮች አሉዎት? አዎ ከሆነ! እዚህ የተጠቀሰው:_________________________________			

10.2	በሆስፒታል ውስጥ ሥራዎን ከጀመሩ በኋላ ሌላ የጤና ችግሮች አጋጥመውታል? አዎ ከሆነ እዚህ የተጠቀሰው:____________________________		
10.3	ላለፉት ወራት ከሥራ ጋር የተያያዘ የጡንቻ ሕመም ገጥሟችኋል?		
#	ለ#10.3፣ "አዎ" ከሆነ በጉዳቱ ምክንያት ስንት ቀን ከስራዎት ቀሩ?		

**4**	**እውቀት (በምርጫዎ ስር የቲክ ምልክት ይጠቀሙ)**	አዎ/የለም
11	በተበከለ ቆሻሻ ምክንያት የሄፐታይተስ ኢንፌክሽን የመያዝ እድልን ያውቃሉ?	
12	በመርፌ ዱላ መጎዳት ከስራ አደጋዎችዎ ውስጥ አንዱ ነው ብለው ያስባሉ?	
13	የሆስፒታሉ ተቋም በጣም ተላላፊ መሆኑን ያውቃሉ	
14	ለሙያ ጉዳት መንስኤ የሆኑትን ሹል እና መርፌዎችን እና መርፌዎችን ታውቃለህ?	
15	በቅንብሮችዎ ውስጥ ስለ የስራ ጤና እና ደህንነት አገልግሎት ያውቃሉ	
16	መርፌዎችን እና ማንኛውንም ስለታም ቆሻሻዎች በጥንቃቄ የማስወገድ ጥንቃቄዎችን ያውቃሉ?	
17	ደህንነቱ ባልተጠበቀ የሥራ ሁኔታ ምክንያት የሥራ ጤና አደጋዎች ሊኖሩ እንደሚችሉ ያውቃሉ	
18	የሆስፒታል ኢንፌክሽን በደም ሊተላለፍ እንደሚችል ያውቃሉ	
19	የሥራ ጫና እና ከመደበኛ አቅም በላይ የሆነ የሙያ ችግር ሊፈጠር ይችላል ብለው ያስባሉ?	
20	የሆስፒታል ኢንፌክሽኖች በሰውነት ፈሳሽ ብክለት ሊተላለፉ እንደሚችሉ ያውቃሉ	

**5.ግንዛቤዎች** 1፡ በጥብቅ፡ 2፡ አልስማማም፡ 3፡ ገለልተኛ አልስማማም; 4፡ እስማማለሁ; 5: በጣም እስማማለሁ)						
21	በሙያ በሽታ የመያዝ እድሌ ትልቅ ነው ብዬ አምናለሁ።	1	2	3	4	5
22	በሙያዬ ውስጥ የስራ በሽታ የመያዝ እድሉ ጥሩ እንደሆነ ይሰማኛል።					
23	በዚህ የሙያ ዘርፍ የሙያ ህመም ያለባቸውን ሰዎች አውቃለሁ					
24	ባዮሎጂካል አደጋን ለመከላከል ከድህረ ተጋላጭነት ፕሮፊላክሲስ አውቃለሁ					
25	እንደ የግል መከላከያ መሳሪያ ያሉ መደበኛ ጥንቃቄዎችን መከተል የሥራውን አደጋ እንደሚቀንስ አምናለሁ።					
26	ስልጠና ከስራ ጤና እና ደህንነት አደጋን ሊቀንስ ይችላል።					
27	በሥራ ላይ ሕመም የማግኘት ሐሳብ በጣም አሳሳቢ ነው					
28	የሥራ ሕመም ካጋጠመኝ ሥራዬ አደጋ ላይ ይወድቃል					
29	ከስራ ጤና እና ደህንነት አደጋዎች ነፃ እንደሆንኩ አምናለሁ።					
30	በስራ ላይ ያሉ የጤና እና የደህንነት አደጋዎች እድላቸው ትንሽ ወይም ትንሽ ነው ብዬ አምናለሁ።					

**6. ለሥራ ጤንነትና ደህንነት ጉዳቶች አባባሽ**		አዎ	የለም
31	ስካሁን የሙያ እና የጤና ደህንነት ስልጠና አግኝተዋል?		
32	ከሥራ ጋር በተያያዙ ችግሮች ምክንያት የእንቅልፍ መዛባት/ረብሻ አለህ?		
33	በአሁኑ ጊዜ አልኮል ትጠቀማለህ?		
34	በአሁኑ ጊዜ የሥራ ጫና አለብህ?		
35	በአሁኑ ጊዜ ተጨማሪ 8 ሰአታት ይሰራሉ?		
36	በአሁኑ ጊዜ ጫት ትመጫለህ?		
37	በአሁኑ ጊዜ ትምባሆ ታጨሳለህ?		
38	የሥራ ጫና አለብህ?		
39	አሁን ባለው ሥራዎ እንዴት ይረካሉ?		

40	የሥራ አካባቢን እንዴት ማርካት ይቻላል?		
41	ለስራዎ ማህበራዊ እውቅና አለ?		
**7.**	**የግል መከላከያ መሳሪያዎች/PPE አጠቃቀም፣ አቅርቦት እና ምቹ (አዎ/አይ)**	**አዎ**	**አይደለም**
42	የግል መከላከያ መሳሪያዎችን ምን ያህል በተደጋጋሚ መጠቀም?		
43	የግል መከላከያ መሣሪያዎች ምቹ ናቸው?		
44	የግል መከላከያ መሳሪያዎችን ምን ያህል በተደጋጋሚ መጠቀም?		
45	የግል መከላከያ መሣሪያዎች ሥራዬን የመሥራት አቅሜን ይረብሹኛል።		
46	የግል መከላከያ መሣሪያዎች ሥራዬን የመሥራት አቅሜን ይረብሹኛል።		
47	የግል መከላከያ መሣሪያዎች ሁልጊዜ ለእኔ አይገኙም።		
48	የግል መከላከያ መሳሪያዎችን መልበስ ለወደፊቱ የጤና ችግሮች በስራ ምክንያት ሊከሰቱ ይችላሉ።		
49	የግል መከላከያ መሳሪያዎችን እኔ በሥራ ላይ ላለሁባቸው አደጋዎች መጋለጥን ይከላከላል		
50	የግል መከላከያ መሳሪያዎችን በሚለብስበት ጊዜ የሙያ በሽታ ስለመያዝ አልጨነቅም		
51	የግል መከላከያ መሳሪያዎችን በመልበስ እጠቀማለሁ።		
52	በየእለቱ ከአስተዳዳሪዬ የተሰጠ ማሳሰቢያ ለኔ የግል መከላከያ መሳሪያ ልበስ ጠቃሚ ነው።		
53	ተቆጣጣሪዬ እኔን ሲመረምር የግል መከላከያ መሳሪያዎችን መልበስን ያሻሽላል		
54	የዲሲፕሊን እርምጃ ስጋት የግል መከላከያ መሳሪያዎችን እንድለብስ አስፈላጊ ነው		
**8**	**የኢንፌክሽን መከላከል እና ቁጥጥር ጥያቄዎች**		
55	በሆስፒታልዎ ውስጥ ኢንፌክሽን መከላከል እና መቆጣጠርን ይለማመዳሉ?		
56	ወደ መደበኛ ንግድዎ ሲሄዱ የግል መከላከያ መሳሪያዎችን ይለብሳሉ?		
57	ቆሻሻ በሚሰበስቡበት ጊዜ እንደ ደም ያሉ የሰውነት ፈሳሾችን ማፍሰስ ይለማመዳሉ?		
58	ስራዎን ከጨረሱ በኋላ እጅዎን በትክክል ይታጠቡ?		
59	ቆሻሻን ከምንጩ ይለያሉ?		
60	የሕክምና ቆሻሻን በአስተማማኝ መንገድ ታስወግዳለህ?		
61	ጥሩ ሳል እና የመተንፈሻ አካላት ንፅህናን ይለማመዳሉ?		
62	ስራህን ሰዎችን በመመገብ ላይ የተመሰረተ ነው?		
63	መርፌዎችን እና አደገኛ ነገሮችን በደህንነት ሳጥን ውስጥ ያስቀምጣቸዋል?		
64	የሕክምና ቆሻሻን በቢጫ መያዣዎች ውስጥ ይጥላሉ?		

አስቀድመህ በጣም አመሰግናለሁ!
