# Supplementary material for: Occupational injuries and associated factors among sanitary workers in public hospitals, eastern Ethiopia: A modified Poisson regression model analysis
Source: PLoS One. 2024 Nov 15;19(11):e0310970. doi: 10.1371/journal.pone.0310970 (PMC11567533; doi:10.1371/journal.pone.0310970)
Supplement: S4 File — (PDF) [file pone.0310970.s004.pdf]

Ulliyada Caafimaadka iyo Sayniska Caafimaadka ee Jaamacadda Haramaya ,
Code:______

A. Nooca luqadda Ingiriisiga ee Dadka Waaweyn ee Kartida leh: Da'da > 18 Sano)

1. **Horudhac**: Magacaygu waa ___________________________________, waxaan u shaqaynayaa xog ururiye ahaan daraasadda ay wadaan bulshadan ay wadaan kooxda Cilmi-baarista (Sina Temesgen Tolera, Tesfaye Gobena, Nega Assefa, Abraham Geremew iyo Elka Toseva). Waxaan si naxariis leh kaaga codsanayaa inaad i siiso dareenkaaga si aan kuugu sharaxo daraasadda iyo in laguu doorto ka qaybqaataha daraasadda.
2. **Ciwaanka daraasadda/mashruuca:** Culayska Dhaawacyada shaqada iyo go'aaminta shaqaalaha nadaafadda ee cisbitaalada dadweynaha ee bariga Itoobiya
3. **daraasadda:**Natiijooyinka daraasaddan waxay noqon kartaa mid muhiimad weyn u leh Isbitaalka iyo qaybaha kale si ay u qorsheeyaan ku dhaqanka caafimaadka iyo badbaadada deegaanka, badbaadada shaqaalaha nadaafadda sida nadiifiyeyaasha, qashin aruuriyaasha iyo kuwa kale. Waxaa intaa dheer in daraasaddani ay qayb ka tahay buuxinta shuruuda laga rabo waxbarashada heerka saddexaad ee Dhakhtarka Falsafadda (*PHD*) barnaamijka Caafimaadka Deegaanka ee shaqsiga hawshan daraasada wada *(principal Investigator)*.
4. **Habka iyo muddada:** Waxaan wareysan doonaa shaqaalaha nadaafadda isbitaalka, sida nadiifiyeyaasha, qashinka aruuriya iyo shaqaalaha bullaacadaha anigoo isticmaalaya xog-wareysi iyo indho-indheyn jireed si ay ii siiyaan xogta muhiimka ah ee caawinta u leh daraasadda. Waxaa jira **96** su'aalood oo laga buuxin doono jawaabaha lawareystaha. Wareysiga shaqaalaha nadaafadda isbitaal kasta wuxuu qaadan doonaa qiyaastii 45-50 daqiiqo.
5. **Khatarta iyo faa'iidooyinka:**Khatarta ka-qaybgalka daraasaddan waa mid aad u yar, laakiin waxay qaadanaysaa daqiiqado yar oo keliya wakhtiga shaqaalaha nadaafadda. Ma jiri doonto lacag toos loogu bixinayo ka qaybqaadashada daraasaddan. Laakiin natiijooyinka ka soo baxa cilmi-baaristan ayaa laga yaabaa inay daaha ka qaadaan macluumaadka muhiimka ah ee cisbitaalka gaar ahaan wax ka beddelka habraaca caafimaadka iyo badbaadada shaqada, taas oo ah qayb ka mid ah ka hortagga caabuqa (*infection)* iyo hawlaha xakamaynta ee isbitaalka.
6. **Xog ilaalin (Qarsoodi):** Macluumaadka nala siin doono waxa ay ahaan doonaan kuwo sir ah. Ma jiri doono macluumaad tilmaamaya gaar ahaan ka qaybgalayaasha. Natiijooyinka daraasadda ayaa noqon doona mid guud oo loogu talagalay bulshada daraasadda mana ka tarjumayso wax gaar ah oo shakhsi ah. Foomka xog-ururinta ayaa koodh loo samayn doonaa si aan loo muujin magacyada. Wax tixraac ah laguma samayn doono warbixino hadal ama qoraal ah oo xidhiidhin kara ka qaybgalayaasha cilmi-baadhista.
7. **Xuquuqda:** Ka-qaybgalka daraasaddan waa ikhtiyaari. Ka qaybgalayaashu waxay xaq u leeyihiin inay caddeeyaan inay ka qaybqaataan ama aanay ka qaybgelin daraasaddan. Haddii ay go'aansadaan inay ka qaybqaataan, waxay xaq u leeyihiin inay ka baxaan daraasadda wakhti kasta tanina kuma keenayso inay lumiyaan faa'iidooyinka ay xaqa u leeyihiin. Uma baahna inay ka jawaabaan su'aal kasta oo aysan rabin inay ka jawaabaan.
8. **Cinwaanka xiriirka:**Haddii ay jiraan wax su'aalo ah ama aad waydiiso wakhti kasta oo ku saabsan daraasadda ama nidaamka, fadlan la xiriir: Sina Temesgen: sinatem3@gmail.com; +251913023634; Guddiga Dib-u-eegidda Anshaxa Cilmi-baarista Caafimaadka ee Hay'adda (IHRERC) xafiiska kala soo xiriir 0254662011 ama P.O.Box 235, Harar, Ethiopia.
9. **Ku dhawaaqida ogolaanshaha ikhtiyaariga ah ee la wargaliyay:**Waan akhriyay/ la ii akhriyay xaashida macluumaadka ka qaybqaataha. Waxaan si cad u fahmay ujeedada cilmi-baarista, hababka, khatarta iyo faa'iidooyinka, arrimaha sirta, xuquuqda ka qaybgalka iyo ciwaanka xiriirka wixii su'aalo ah. Waxaa la i siiyay fursad aan ku weydiiyo su'aalaha aan ii caddayn. Waxaa la igu wargeliyay inaan xaq u leeyahay inaan ka baxo daraasadda wakhti kasta ama inaan ka jawaabin su'aal kasta oo aanan rabin. Sidaa darteed, waxaan caddaynayaa oggolaanshahayga ikhtiyaarka ah si aan uga qaybqaato daraasaddan oo leh xarfaha hore (saxiixa).

Magaca iyo saxeexa ka qaybqaataha: ____ Taariikhda____________

Magaca iyo saxeexa xog ururiyaha: ____ Taariikhda _____________

Xusuusnow

- Tan waxaa loo saxiixay weji ka waji iyadoo uu joogo xog aruuriyaha.
- Fadlan sii koobiga ogolaanshahan saxeexan ka qaybqaataha.
- Haddii ka qaybqaataha uu yahay qof caadi ah oo uusan saxiixi karin xarfaha hore, wuxuu ku dhejin karaa suulka hortiisa markhaati karti u leh; maragguna waa inuu saxeexaa (magacooda iyo ciwaankooda).

n.t	1.Astaamaha tirakoobka bulshada (Xeerka ka qaybqaataha_____________)		
1.	Shaqaalaynta: 1 Joogto ah 2. Qandaraas 3. Dibadda laga keenay 4.kale		
2.	Jinsiga | Lab | Dheddig		
**3**	Da'da ___________		
**4**	Khibrada Shaqo __________		
**5**	Heerka waxbarasho ___________		
**6**	Xaalada Guurka: Kali Guursaday Kala maqan La furay		
**7**	Mushaharka bishii: __________		
**8**	Qaybaha shaqada Nadiifiyeyaasha Qashin-qaadayaasha Qashinka faaruqiyay		
	Nooca shaqadaada: Shaqada 1aad ____ shaqada 2aad ____shaqada 3aad______		
	**2.Dhaawacyada Caafimaadka Shaqada**		
**9**	Miyaad ku dhaawacantay 12 bilood ee la soo dhaafay dhaawacyo la xiriira shaqada?	Haa	Maya
	Haddi "HAA" #09, waa maxay inta jeer ee dhaawaca shaqada sanad gudihiis?<br>Hal mar Laba jeer In ka badan laba		
	Haddii "HAA" #09, waa maxay Nooca/ Noocyada dhaawaca? (Sax[√] Hal ama ka badan		
	1. Murug (waxaa ku jira nabar)<br>2. Guud ahaan Goynta<br>3. xoqid jirka<br>4. Nabar-goyn/Goyn qallafsan/<br>5. Jeex-jeexid/Gooyn qoto dheer<br>6. Kala-bax/<br>7. Daloolin<br>8. Jab<br>9. Xasaasiyadda iyo isxoqa, (maqaarka, isha,cabudh)<br>10. Goynta (Nooca jirka ___________)		
	Haddi "HAA" #09, oo "Qayb/qaybaha jidhka dhaawacmay [Sax[√] hal iyo wax ka badan hal)		
	Faraha Gacmaha Lugaha cag/suul<br>Madaxa Ilkaha Indhaha Wax kale__________		
	Haddii "HAA" #09, maxay ahaayeen sababaha dhaawaca? [Sax[√] hal iyo wax ka badan hal]		
	1. Dhaawac fiiqan ama irbad<br>2. kasoo dhicid meel<br>3. Qalabka gacanta<br>4. Siibasho<br>5. wax Ku soo dhacay<br>6. Kala qaybinta qashinka<br>7. U adeegsiga si xun/aqoon daro qalabka ilaalada shaqsi<br>8. La dagaallanka asxaabtaada/madaxdaada/		
	Haddii "HAA" #09, imisa maalmood oo shaqo ayaa ku luntay dhaawacyo? ___Maalmo		
	**3)Cudurada la xiriira shaqada ama naafanimada**	Haa	Maya
**10.1**	Kahor intaadan ka bilaabin shaqada cusbitaalka, majirtay dhibaato kale oo caafimaad daro? Hadday haa tahay! Halkan ku xus :____		
**10.2**	Kadib markaad shaqada ka bilowday cusbitaalka, ma jirtay dhibaato kale oo caafimaad daro kulasoo daristay? Hadii Haa tahay. Halkan ku xus:_____________________________		
**10.3**	Ma kala kulantay shaqada xanuunka murqaha 12kii bilood ee la soo dhaafay?		
#	Haddii "HAA" #10, imisa maalmood oo shaqo ayaa ku luntay dhaawacyo? ___Maalmo		
	**4. Aqoon**	Haa	Maya
11	a garanaysaa fursada infekshanka cagaarshowgu uu sababo qashinka wasakhaysan?		
12	Ma kula tahay dhaawaca ul irbadda inuu yahay mid ka mid ah khatarahaaga shaqo?		
13	Ma ogtahay in xarunta cisbitaalku aad u faafayo		
14	Ma garanaysaa qaybo fiiqan iyo irbado iyo irbado sababaha dhaawaca shaqada?		
15	Ma ka taqaan adeegga caafimaadka iyo badbaadada shaqada ee goobahaaga		
16	Ma garanaysaa taxaddarrada badbaadada leh ee cirbadaha iyo qashinka fiiqan?		
17	Ma ogtahay in khataraha caafimaadka shaqadu ay ugu wacan tahay xaaladaha shaqada ee aan badbaadada lahayn		

18	Ma ogtahay in infekshannada isbitaallada lagu kala qaado dhiigga				
19	Ma u malaynaysaa in culayska shaqada iyo wixii ka baxsan awoodda caadiga ah ay keeni karaan dhibaatooyin xagga shaqada ah?				
20	Ma ogtahay in infekshannada nosocomial lagu kala qaado wasakhowga dareeraha jidhka				
	**5 Aragtida: Halkee: 1:aad u adag, 2: khilaafsan, 3: Dhexdhexaad Diid; 4: Ogow; 5:Aad baan ugu raacsanahay)**	1	2	3	4
21	Waxaan rumaysanahay in fursadahayga ah in uu igu dhaco jirro shaqadu ay aad u wayn tahay				
22	Waxaan dareemayaa in aan fursad fiican u haysto in aan ku dhaco jirro shaqada shaqadayda				
23	Waxaan aqaan dadka ku jira shaqadan xirfadeed oo qaba jirro shaqo				
24	Waan ka warqabaa kahortaga soo-gaadhista ka dib si looga hortago khatarta noolaha				
25	Waxaan aaminsanahay in raacitaanka taxaddarrada caadiga ah sida PPE ay yarayn karto khatarta shaqada				
26	Tababarku wuxuu yarayn karaa khatarta caafimaadka iyo badbaadada shaqada				
27	Fikirka in uu ku dhaco jirro shaqadu aad bay u khusaysaa				
28	Haddii aan igu dhaco xanuun xagga shaqada ah, xirfadayda khatar bay geli lahayd				
29	Waxaan rumaysanahay in aan ka xoroobay khataraha caafimaadka iyo badbaadada shaqada.				
30	Waxaan rumaysanahay in fursadaha khataraha OHS ay yihiin kuwo yar ama xitaa aan macno lahayn				
	**6. Arrimaha isku xidhan ee Dhaawacyada Shaqada**				
31	Weli ma heshay tababar badbaadada shaqada iyo caafimaadka?	Haa	Maya		
32	Ma qabtaa hurdo la'aan/qalalaas ka dhashay dhibaatooyinka shaqada la xiriira?				
33	Hadda, ma isticmaashaa khamri?				
34	Hadda ma haysaa culays shaqo?				
35	Hadda ma shaqeysaa in ka badan 8 saac/maalintii?				
36	Hadda ma cuntaa qaadka?				
37	Hadda ma sigaar cabtaa?				
38	Ma qabtaa culays shaqo?				
39	Sideed ugu qanacsan tahay shaqadaada hadda?				
40	Sideed ugu qanacsan tahay jawiga shaqada?				
41	Ma jiraa aqoonsi bulsho oo shaqadaada ah?				
	**7.Qalabka Ilaalinta Shakhsi ahaaneed/Ka faa'iidaysiga PPE, Bixinta iyo raaxada (Haa/Maya)**	**Haa/Maya**			
42	Intee la'eg isticmaalka qalabka ilaalinta shakhsi ahaaneed?				
43	Qalabka Ilaalinta Shakhsiyeedka ma ku fiican yahay?				
44	Intee la'eg isticmaalka qalabka ilaalinta shakhsi ahaaneed?				
45	Qalabka ilaalinta shakhsi ahaaneed ayaa farageliya awooddayda aan ku qabsan karo shaqadayda				
46	Qalabka ilaalinta shakhsi ahaaneed ayaa farageliya awooddayda aan ku qabsan karo shaqadayda				
47	Qalabka ilaalinta shakhsi ahaaneed mar walba ma heli karo				
48	Xidhashada PPE waxay iga ilaalin doontaa mustaqbalka dhibaatooyinka caafimaadku waxay ku dhici karaan shaqada awgeed				
49	PPE waxay ka hortagtaa soo-gaadhista noocyada khatarta ah ee aan ku suganahay shaqada				
50	Kama werwero in aan ku dhaco jirro shaqo marka aan xidho qalabka ilaalinta shakhsi ahaaneed				
51	Waxaan ka faa'iidaysanayaa xidhashada qalabka ilaalinta shakhsi ahaaneed				

52	Xusuusinta kormeerahayga maalin kasta waxay muhiim u tahay xidhashada qalabka ilaalinta shakhsi ahaaneed		
53	Kormeerahayga oo i eegaya waxa ay hagaajin doontaa xidhashada qalabka ilaalinta shakhsi ahaaneed		
54	Khatarta falka edbintu waa arrin muhiim ah oo lagu hubinayo in aan xidho qalabka ilaalinta shakhsi ahaaneed		
	**8.Su'aalaha ka hortagga iyo xakameynta caabuqa (Haa/Maya)**	**Haa/Maya**	
55	Ma ku shaqeysaa ka hortagga iyo xakameynta caabuqa isbitaalkaaga?		
56	Miyaad xidhataa qalabka ilaalinta shakhsi ahaaneed markaad samaynayso ganacsigaaga caadiga ah?		
57	Ma ku celcelisaa daadinta dareerayaasha jirka, sida dhiigga, markaad qashinka ururinayso?		
58	Si sax ah ma u dhaqdaa gacmahaaga kadib markaad dhameysato shaqadaada?		
59	Ma kala saartaa qashinka meesha?		
60	Ma u daadisaa qashinka dawada si badbaado leh?		
61	Ma ku dhaqantaa qufac wanaagsan iyo nadaafadda neefsashada?		
62	Ma ku salaysan tahay shaqadaada dhaqanka quudinta dadka?		
63	Ma ku haysaa cirbadaha iyo walxaha khatarta ah sanduuqa badbaadada?		
64	Ma ku shubtaa qashinka dawada weel huruud ah?		

Aad baad ugu mahadsantahay horay!
