# Supplementary material for: Occupational injuries and associated factors among sanitary workers in public hospitals, eastern Ethiopia: A modified Poisson regression model analysis
Source: PLoS One. 2024 Nov 15;19(11):e0310970. doi: 10.1371/journal.pone.0310970 (PMC11567533; doi:10.1371/journal.pone.0310970)
Supplement: S5 File — (PDF) [file pone.0310970.s005.pdf]

B. Key Informant Interview (IPC experts) For Competent Adults: Ages > 18 Years Code:______

My name is ___________________________________, I am working as a data collector for the study being conducted in this community by Research team (Sina Temesgen Tolera, Tesfaye Gobena, Nega Assefa, Abraham Geremew and Elka Toseva). I kindly request you to lend me your attention to explain you about the study and being selected as the study participant.

NB

Dear! **It self-administered ,** thus, you should evaluate the following possible causes of occupational injuries based on your skill knowledge and experience you have in the hospitals. The following criteria help you to evaluate provided item

1) **Severity(S)** is the impact of the events, categorized as slight effect ranged from 1 to 5 (Score: 1), minor severe with absence illness (2), moderate injuries requiring hospital admission (3), major injuries and illness in permanent (4) and death (5).
2) **Exposure** (E)is the frequency of events ranged from 1 to 5, categorized as very rarely, year (Score=1) monthly (2), weekly (3) and daily (4), constantly, multiple times a day (5).
3) **Probability** is the likelihood of the events ranged from 1 to 5, categorized as chance to occurs is not expected through life of the work (Score=1), it indicates chance to conceivable is between 10-30 of working years (2), chance between 1-10 working years (3), chance within a month (4), and chance to occurs daily to weekly (5).

S.no	Possible causes of Occupational injuries in hospitals	**SEP**		
Q1	Occupational injuries due to improper medical waste management at sources	**S**	**E**	**P**
Q2	Occupational Injuries due to needle stick, broken glass, plastic and sharp materials			
Q3	Occupational burns due to chemical detergents and solvents and hazardous exposures			
Q4	Occupational problems due to inappropriate utilization of PPE			
Q5	Occupational problems due to insufficient personal protective equipment/PPE			
Q6	Occupational problems due to lack of safety training for sanitary workers			
Q7	OHS problems due to exposure to poor recognition for sanitary workers			
Q8	Occupational injuries due to poor transportation of medical waste management			
Q9	Develop occupational illness due to lack of post exposure prophylaxis			
Q10	Exposure to biological hazards like HIV/AIDS, Hepatitis B and other pathogens			
Q11	OHS problems due to poor practice of OHS and lack of its guideline			
Q12	Occupational health and safety outcomes due to poor IPC member support			
Q13	Occupational injuries due to poor passageway for transportation of medical waste			
Q14	Occupational Injuries due to fall, slip, hit, caught equipment and materials			

C. Key Informant Interview [Sanitary Workers Representative] Code: ____________________

(For Competent Adults: Ages > 18 Years)

Dear/madam!

My name is __________________________________, I am working as a data collector for the study being conducted in this hospital by Research team (Sina Temesgen Tolera, Tesfaye Gobena, Nega Assefa, Abraham Geremew and Elka Toseva). I kindly request you to lend me your attention to explain you about the study and being selected as the study participant. This interview contains about 15 questions with "YES/No" response and then need some justification with existence and reality within your setting. This might be taking 40-50 minute.

If so can I start? Thank you!

Interviewer contact information

Name of setting: _______________________________________

Email address of interviewer: _______________________________

Date of key informant interview: ______/______________/_______

If any Information at beginning ____

B. Key informant contact information

Name of key informant: ____

Title of key informant: ____

Address of key informant: ____

Email of key informant: ____

Contact phone number: ____

General background information

1. General OHS Guidelines

Is there guideline and policies for hospital sanitary workers regarding the followings? Yes/No

If No what do think?

____

____

____

Is there an occupational safety and health policy endorsed by management in the facility?

2. Occurrence of occupational related injuries

Have you seen when occupational related problems happened? Is there reporting system? No/Yes

How you were managed that?

____

____

____

____

3 Training for Cleaners

How frequently do cleaners and other personnel directly involved inpatient care receive training regarding IPC in your facility? Yes/No

If No why?

Is there training schedule for cleaners? Yes/No

If No why?

4. Adequate Supervision

Is there adequate supervisor schedule for cleaners? Yes/No

If No why??

Do you supervise their utilization? Yes/No

If No why?

5. Job rotation for Cleaners

Do cleaners or sanitary workers have job rotation? Yes/No

If Yes, do though as job rotation could reduce occupational health and safety problems?

No, why didn't?

6. Availabilities of PPE

Are there available of PPE (Glove, Mask, Boots, Goggle, and apron/Gown)? Yes/No

If No why?

7. Conformability of PPE

Are there comfortable? (Glove [Surgical Heavy duty] , Mask, Boots, Goggle, apron/Gown ; Yes/No

If No why?

8. PPE Utilization

Do you think cleaners/ sanitary workers used PPE properly? Yes/No

If No why?

9. Post exposure Prophylaxis /PEP/

Is there Screen for HBV? Yes/No and for HIV/AID? for the cleaners? Yes/No

Is there available of PEP (It should be must for Hepatitis B and for other injuries) Yes/No

If No why? ______________________________

10. Confined Room for workers

What facilities currently exist for cloth changing room for both male and female? Yes/No
If no why?

____
____
____

11. Confined Space for PPE

What do you think about utilization of PPE among cleaners/ sanitary workers? Yes/No
If No why?

____
____
____
____

12. Hand hygiene after they are finalized their work

Is hand washing soap available in the hospital and do cleaners use it? Yes/No If No why?

____
____

13. Well design path/rood/ for materials/Waste transportation up to final

Do you thinking the road, infrastructure and size of materials they are moving (Water bald, waste bin safe for cleaners working? Proximal/Distal or Stretch/exertions
Yes/No____
____
____
____

Do you think improper moving or bad infrastructure are one of the causes for occupational health and safety? Health problems? Yes/No
If "No" why?

____
____
____

14. OHS Guidelines/Law from Government/Partner Support

Regarding the followings, are there any policies or laws, or any government programs in place to support Hospital sanitary workers including others?

13.1 Is OHS Guidelines for "Personal protective Equipment" in your setting?
Yes/No
Frequency of distribution per a year? A) Once or B) Twice C) triple? [In case]
At what month per a year? ____

13.2 Is there "First Aid while workers injured" in your settings?
Yes/No

____

13.3 Is there "Medical test/Screening Hepatitis B virus and HIV/AIDS" in your settings?
Yes/No
Frequency of screening per a year? A) Not given still, B) Once or C) Twice D) triple
At what month per a year? ____

13.4 Is there "Occupation Safety injuries/Incidence reporting" in your settings?
Yes/No
If Yes, By whom ____

13.5 Do have" hazard/Risk Prioritization" in your setting?
Yes/No

By whom ____
Do know the concept? If yes explain ______________________________________

15. General Medical Waste management
How you evaluated your medical waste management from source to disposal? **Good/Bad,** observe [Take photo]
Is the waste are segregated? **Yes/No** [Take photo]
16 General Infection Prevention and Control Implementation
Do have IPC teams? **Yes/No, I don't know**
Yes "Yes" Are you one of the IPC members? **Yes/No**
____
Are top management team supporting sanitary works? **Yes/No**, if "Yes" How?
____
Are top management team aware about OHS service in IPC activities? **Yes/No.**
____
____
Are top management team supporting sanitary workers? Yes/No. If yes How?
____
____
____

D. Field Observation

i. Utilization of PPE among sanitary workers

____
____

ii. Hospital waste management

____
____
____
____

iii. Possible cause of occupational injuries

____
____
____

Thank you very much for your response and time!
