# Supplementary material for: Occupational injuries and associated factors among sanitary workers in public hospitals, eastern Ethiopia: A modified Poisson regression model analysis
Source: PLoS One. 2024 Nov 15;19(11):e0310970. doi: 10.1371/journal.pone.0310970 (PMC11567533; doi:10.1371/journal.pone.0310970)
Supplement: Attachment — Submitted filename: Reviewers Comment.pdf [file pone.0310970.s007.pdf]

**REVIEWER'S COMMENTS**

**Abstract**

- Line 33: "Data were collected from 809 from hospital sanitary workers" should be read as "Data were collected from 809 hospital sanitary workers"
- Line 34: The occupational injury….., "The" should be deleted.
- Lines 36 – 38: "Binary and multiple logistic regressions were used to explore the relationship between dependent and independent variables. The crude odds ratio (COR) and adjusted odds ratio (AOR) with a 95% confidence interval (CI) were used to present the analysis." Binary logistic regression reports crude ratio and multiple logistic regression also reports adjusted odds ratio so it is not necessary to include "The crude odds ratio ……..". Authors can add "Statistical significance was determined at a 95% confidence level" after "…………. "between dependent and independent variables".
- Line 39: Authors indicated in the methods section of the abstract that 809 respondents were used for the study. However, in the results section of the abstract, they indicated 729. The authors should clarify the inconsistency.
- Lines 41 – 42: Authors should indicate the unit of measurement of age and work experience.
- The results section is not very clear. Authors should consider re-writing the results to make them clearer.

**Introduction**

- Line 65: "They often cited" should be written as "They are often cited".
- Lines 69 – 70: This needs to be revised to make it clearer.
- Line 73: "but also they are facing with a variety of…" should be written as "but also they face several occupational ….".
- Line 97: Reference (35) should be in square brackets, [35].

**Methods**

- Line 109 – 111: "In terms of study areas, public hospitals within a 200km radius of Harar were chosen from three regions and one administrative city". This statement is not clear. The authors should justify why they selected the study areas.
- Lines 114 – 117: "From ….." the content should be checked and it is a bit unclear.
- Line 115: The word "daily" should be deleted.
- Lines 116 – 117: The (mean+SD) values in the bracket are 4 but the parameters are three (bed occupancy, outpatient flow and inpatient). This should be rectified.
- Line 117: "Out of 5680 hospital staff of 5680" This statement should be checked as 5680 is repeated.
- The map of the study area is blurred. Authors should replace it with a clear map.
- Line 122: Section 2.2 "Source and study population" should be revised to read "Study population".
- Line 134: The sentence is incomplete.
- Line 135: Section 2.4 - Sample determination. The entire section is not clear. Authors should be specific on how they estimated the sample size for the study.

- Line 151: Section 2.5 Selection Procedures should be written as "Sample selection procedures". Authors should explain how they arrived at the sample size for each of the four study States.
- Line 165: About Figure 2. According to the information in section 2.5, Dire Dawa City has a total sample size of 82. However, it says otherwise in the Figure. The same applies to the Oromia Regional state.
- Line 175: "………workers' representatives (KII-2) were purposely selected from each eight". The authors should check this statement again as there may be an error with the use of "eight".
- Lines 192 – 194: Authors should check the time shifts as they overlap and create confusion for readers.
- Lines 228 – 246: The information presented here does not form part of quantitative data. Key informant interviews are ways of collecting qualitative data. Authors should kindly revise.
- Line 258: "independent variable" instead of "variable independent"
- Lines 267 – 274: Section 2.10 Ethical statement. Authors should take the entire section out.

**Results**

- Lines 288 – 289: "Of these, 43.59% (95%CI: 39.87, 47.42) and 50.00% (95%CI: 35.52, 64.47) were cleaners and solid waste collectors, respectively". The percentages of cleaners and solid waste collectors should sum up to 100%. This needs to be cross-checked.
- Table 3: Authors should provide a note under the table to explain the meaning of values with single asterisks.
- Line 330: Section 3.6 – Assessment from IPC as KII. This section per the methods is supposed to be qualitative. However, nothing shows under the results section. Authors should present their qualitative findings.

**Discussion**

- The discussion could be improved by revising the qualitative aspect to reflect the suggested revision in the results section.
- Authors should only discuss the findings based on the objectives of the study and delete any other thing that is not related to the objectives of the study.
- The authors could improve the discussion by including the implications of the findings.

**Conclusion**

- This will have to be reconsidered after effecting all the suggested corrections.

**General comment**

- There are many errors in the manuscript that need to be corrected. I suggest authors proofread the entire manuscript. Some parts have been highlighted yellow and green

and I do not know the rationale for doing that. Authors should ensure they submit a clean document.

- A few mentions of the errors can be found in lines 53, 102, 103 115, 138 – 140, 192, 248 – 249, 256, 472, 473 – 474, and 486 – 487.
