# Supplementary material for: Occupational injuries and associated factors among sanitary workers in public hospitals, eastern Ethiopia: A modified Poisson regression model analysis
Source: PLoS One. 2024 Nov 15;19(11):e0310970. doi: 10.1371/journal.pone.0310970 (PMC11567533; doi:10.1371/journal.pone.0310970)
Supplement: Attachment — Submitted filename: Response to Reviewers.pdf [file pone.0310970.s008.pdf]

June, 2024

Subject: Sending revised manuscript, "**PONE-D-23-41265**"

Greetings to

- ⇨ PLOS ONE Editor
- ⇨ PLOS ONE Editorial team
- ⇨ Reviewers

Kindly, as corresponding Author, on behalf of the rests, I would start by extending a warm greeting to the PLOS ONE's chief editor, the editorial team, and the reviewers.Please allow me to express my gratitude to the PLOS ONE editors and reviewers for your sincere advice, criticisms, suggestions and recommendations. These contributions and efforts are very significant to the quality of our main manuscript in the current version.

Having this, we have used the following format of table to address all issues forward from reviewers (Reviewer 1 and Reviewer 2) ,which embedded in the attached table. Within a manuscript, we used or highlighted with two colors: "Yellow"used for modification and "Cyan Green" used for newly added paragraphs addressed in "Revised Manuscript with Track Change"

Some comments on Knowledge , Attitude, Personal protective equipment and Infection prevention questions were raised. For the mentioned items, the questions were prepared as associated /determinants of occupational injuries, which are submitted as supplementary material. The response for each categorical variables designed to use for other, despite of fact that we used them as summary by giving cut point for each variable., which explained in the method section

S.no	Editor and Reviewers' (R1,R2 and R3) comments	Authors' feedback
	Academic Editor	
A	When submitting your revised manuscript:<br>• A rebuttal letter that responds to each point raised by the academic editor and reviewer(s). You should upload this letter as a separate file labeled 'Response to Reviewers'.<br>• A marked-up copy of your manuscript that highlights changes made to the original version. You should upload this as a separate file labeled 'Revised Manuscript with Track Changes'.<br>• An unmarked version of your revised paper without tracked changes. You should upload this as a separate file labeled 'Manuscript'	Once again, really, we appreciating all the comments, suggestions and recommendations for our paper. In the current form of the manuscript is very attractive and easily understandable due to your huge efforts, having this, these [3 points are done] as suggestions
1	When submitting your revision, we need you to address these additional requirements.<br>Please ensure that your manuscript meets PLOS ONE's style requirements, including those for file naming.	Thank you , as far as our concern, and check PLOSONE guideline, we tried to inline our manuscript with it.
2	Please provide additional details regarding participant consent. In the ethics statement in the Methods and online submission information, please ensure that you have specified (1) whether consent was informed and (2)	• It done and stated including ethical number under methods section [**Line 300-304**]

	what type you obtained (for instance, written or verbal, and if verbal, how it was documented and witnessed). If your study included minors, state whether you obtained consent from parents or guardians. If the need for consent was waived by the ethics committee, please include this information.<br><br>If you are reporting a retrospective study of medical records or archived samples, please ensure that you have discussed whether all data were fully anonymized before you accessed them and/or whether the IRB or ethics committee waived the requirement for informed consent. If patients provided informed written consent to have data from their medical records used in research, please include this information	• It is not medical records
3	We note that your Data Availability Statement is currently as follows: [All relevant data are within the manuscript]. Please confirm at this time whether or not your submission contains all raw data required to replicate the results of your study.	It as been stated that All relevant data are within the manuscript", which is addressed (**line 578**) . If important , we also sent the supplementary data ,in which Adjusted and Un-adjusted Prevalence ratio is extracted
4	Please amend the manuscript submission data (via Edit Submission) to include author Tesfaye Gobena, Nega Assefa, Abraham Geremew and Elka Toseva.	Thank you it is done
5	Please amend either the abstract on the online submission form (via Edit Submission) or the abstract in the manuscript so that they are identical.	Thank it is done
6	Your ethics statement should only appear in the Methods section of your manuscript. If your ethics statement is written in any section besides the Methods, please move it to the Methods section and delete it from any other section. Please ensure that your ethics statement is included in your manuscript, as the ethics statement entered into the online submission form will not be published alongside your manuscript.	Ethics statement has been written in any section besides the Methods
	Additional Editor Comments:	
1	Please include copy of all questionnaires – in original language -- that you used as supplementary materials.	Thank you, it is attached as suggestions
2	Translate key questions that you asked, such as about the outcomes and report in text; Saying that Boolean logic was used is both not adequate and not necessary if you give actual question and response options.	Occupational injuries is our outcome, which is defined in line **171-179** as well as detail in **Line 75-88**
3	Report all percentages to with precision of 1/10, not 1/100, such that 97.29% should be replaced with 97.3%/ Make correction in text and tables. You simply do not have information with precision of 1/100 of 1% when you have <1000 subjects.	All reporting system by one decimal are addressed through the text according to the suggestions
4	Report all COR and AOR (adjusted and unadjusted odds ratios) within 1 decimal place: e.g., 3.6, not 3.61. Make corrections to text and tables throughout.	In this version, we used Un-adjusted and adjusted Prevalence ratio, which are reported as suggestion with one decimal and all Tables according to the suggestions
5	For "mean ± SD for age, work 41 experience, educational status, and monthly income" please report precision within one decimal place or less.	Thank you: It is corrected
6	You cannot conclude that "In order to reduce these risks, appropriate work-related policy relating 53 occupational health and safety be enacted. Further, enforce the implementation of occupational health 54 and safety	Thank you for your feedback. The statement is modified. Despite, the compliance of OHS also evaluated by Hospital experts as well as sanitary workers' representative. Taking this information, we advised to both hospitals and government

	practices within hospitals is necessary." The reason for this is that you did not evaluate any interventions. Please remove all such claims from conclusions but feel free to speculate on plausible interventions in the discussion, esp. if there is evidence to support some specific interventions.	should implement and monitor the OHS service within hospitals, respectively
7	Among keywords, "determinants" is too vague. Determinants of exposure, outcome, something else? Please revise.	Thank you it is corrected as "Determinants of injuries"
8.	Table 3: round up all p-value to within 1 significant figure. Do not highlight values <0.05 with a *, because it is not appropriate to stress null hypothesis testing in observational studies, where non-random errors dominate.(Wasserstein and Lazar 2016)	Thank you for the feedback. Now, the P-values are corrected based on rule of statistics
9.	The rate of self-reported occupational injuries was rather high, 44%, which makes logistic regression a poor tool for approximating relative risk. For OR to approximate RR, the rate of events should be in the range of 10% or less. A more appropriate method of data analysis is to estimate prevalence ratios (PR) using any number of available approaches. In STATA, modified Poisson regression (Zou 2004)is a sensible (better) method. I strongly recommend all analyses be re-done using PR.	Really, we would like to appreciate your excellent feedback for our work. We appropriately used suggested "Modified Poisson regression" Once again thank you very much.
	Reviewers	
1.	Is the manuscript technically sound, and do the data support the conclusions?	
	Reviewer #1: Yes	Thank you very much for your encouragement
	Reviewer #2: Partly	
	Reviewer #3: Yes	
2.	Has the statistical analysis been performed appropriately and rigorously?	
	Reviewer #1: Yes	Thank you very much for your encouragement
	Reviewer #2: Yes	
	Reviewer #3: Yes	
3.	Have the authors made all data underlying the findings in their manuscript fully available?	
	Reviewer #1: Yes	Thank you very much for your encouragement
	Reviewer #2: Yes	
	Reviewer #3: Yes	
	4. Is the manuscript presented in an intelligible fashion and written in standard English?	
	Reviewer #1: Yes	Thank you all for input
	Reviewer #2: No	
	Reviewer #3: No	
	Reviewer #1: Reviewer comments	
	Abstract, line 33 delete from after sample size 809	Thank you, it is corrected
	Introduction<br>Line 60, what do you mean collect solid garbage? Is there liquid garbage?	We corrected as "solid waste"
	Method<br>Line 117 what is mean Out of 5680 hospital staff of 568, about 809 of these were cleaners and waste collectors? What is your justification to take all sanitary workers as a sample? Why you no need sample among sanitary workers?	We used as background of the study. 5680 staffs are those working in selected public hospitals including sanitary workers. But, our plan was only for those staffs their works are associated with cleaning and waste disposing . These are accounted 809 . Despite the statement is improved

<table>
<tr><td></td><td>Line 135, If you took all sanitary workers, what is the need of sample size determination part?? Justify it?</td><td>This section is revised based mother document (Dissertation). Thus, the design effect =2 was added in this version</td></tr>
<tr><td></td><td>Line 121. The map of your study area needs scale, and proper naming of figure is required</td><td>The map is changed considering city where the study areas (hospitals) found</td></tr>
<tr><td></td><td>Line 151. 2.5 selection procedure. There is no proportional allocation, just you took the whole population? What is the need of stating sample selection?</td><td>This section was fully revised including entire figure 2 based on suggestion (line 144-149)</td></tr>
<tr><td></td><td>Line 153, they are not selected?</td><td></td></tr>
<tr><td></td><td>Line 172 your study period was from May 30th, 2023 to August 30th, 2023, why the title of figure 2 is 2022?</td><td>Thank you, its corrected “2023”</td></tr>
<tr><td></td><td>Line 175 unfinished sentence</td><td>Modified (Line 224-226)</td></tr>
<tr><td></td><td>Line 181. 2.6.2 Independent Variables, what is the difference between Institutional variables and Working environment variables? May be enabling variables?</td><td>We revised this section and added both together (Line 207-214)</td></tr>
<tr><td></td><td>Line 202 contents of the questionnaire not question?</td><td>Thank you, it modified as suggested (line 198)</td></tr>
<tr><td></td><td>Line 208 the 7 days Boolean logic questionnaire not mentioned in your abstract of line 34</td><td>It is added per as suggestion (line 39)</td></tr>
<tr><td></td><td>Line 210, what are Determinants questions: eighteen standard questions were prepared for associated factors mean? Are they different from other independent variables?</td><td>In our work, we used Determinants or independent variables or associated factors exchangeable.. Now, it changed into “Associated factor”</td></tr>
<tr><td></td><td>Line 228 Key informant interview = Is it data collection or analysis part? Justify why you need to summarize like quantitative data?</td><td>Thus, we used some of them were for quantitative and s some of them qualitative. As you see some data converted into quantitative in order to compiled the data for easily understanding.</td></tr>
<tr><td></td><td>Line 247. What are the languages used for data collection?</td><td>Three languages: Somali, Oromo and Amharic, which are stated in data quality local language (Line 263-264)</td></tr>
<tr><td></td><td>Line 262, what is MLR?</td><td>Thank you:MLR meant that multiple logistic regression, but In this version it was changed into modified Poison regression</td></tr>
<tr><td></td><td>Line 282 Table 1 Sociodemographic table: Is there separate division of labour for cleaners and waste collectors in the Ethiopian hospital human resource structure? Waste collectors from municipality or have</td><td>Of course, yes. Waste collectors are recruited in some of hospitals due to large of solid waste produced in hospitals.</td></tr>
<tr><td></td><td>Line 288, Burden of occupational Injuries, the calculation and narration is not clear?</td><td>In this version it is more illustrated in Line 309-316 .Despite the burden of occupational injuries calculated as:
1) 43.59% was for only cleaners in the hospitals
2) 50.00% was for waste collectors in the hospitals
3) 44.03% was both of them
<table>
<tr><td>Occupational injuries in the last 12 mont</td><td>Job categories
1. Cleane</td><td>2. Waste</td><td>Total</td></tr>
<tr><td>0</td><td>383
56.41</td><td>25
50.00</td><td>408
55.97</td></tr>
<tr><td>1. Yes</td><td>296
43.59</td><td>25
50.00</td><td>321
44.03</td></tr>
<tr><td>Total</td><td>679
100.00</td><td>50
100.00</td><td>729
100.00</td></tr>
</table></td></tr>
</table>

	Line 310, where do you get neutral attitude?	Attitude toward workplace risk was measured by likert scale (Assessment questions attached as supplementary material). The items then classified unfavored if socre <median(3.00) , neutral if score is 3.0 and favoured media is >3.00 **(Line 210-213**)
	Table 3 and Table 4, If you ran logistic regression why you need to calculate X2 for comparison, please justify?	Table 3: Research team also agreed to delete Table 3 due to less significant for the readers
	Table 4, Variables: Had MSD and conduct supervision are they significant?	According TO current analysis, those had MSD is contribution for likelihood of occupational Injuries at un-dajusted prevalence ratio(**Line 322**) while, lack of supervision had more likely to increase the risk of occupational injuries at adjusted prevalence ratio(**line 336**)
	Line 321. How do you measure Knowledge, attitude and practice as shown in your Table 4?	Knowledge, attitude and practice were measured with standard tools. All cut point of other tools are illustrated (**Line 207-234**)
	Line 330. 3.6 Assessment from IPC as KII, the analysis and result for Key Informant Interview is not clear please make it clear as qualitative data finding?	Thank you. KII well explained in line **236-256.** Regarding their feedback **,**some of KII responses were changed into quantitative data in order to summarize rather explaining for each KII. Thus, average of their feedback was used and the key information was narrated for the readers (**Line 357-370**).
	Line 465, strength, the study had no sample, instead all populations assessed	Thank you very much it is corrected as suggestion **(Line 547**)
	Line 480, Conclusion. You don't have a variable indicating unsafe hygiene, how do you conclude in the first	Thank you: It modified **(Line 562-567**)
	Line 484. Have you addressed the rules, policies and regulations related to OHS?	Thank you for your information. We removed from conclusion and added in the discussion with appropriate meaning (**Line 391-39**3).
	Reviewer #2:	
	Abstract<br>• Line 33: "Data were collected from 809 from hospital sanitary workers" should be read as "Data were collected from 809 hospital sanitary workers"	Thank you very much, it is corrected per a suggestion **(line 35-37)**
	• Line 34: The occupational injury….., "The" should be deleted.	Thank you it is corrected **(Line 39**)
	• Lines 36 – 38: "Binary and multiple logistic regressions were used to explore the relationship between dependent and independent variables. The crude odds ratio (COR) and adjusted odds ratio (AOR) with a 95% confidence interval (CI) were used to present the analysis." Binary logistic regression reports crude ratio and multiple logistic regression also reports adjusted odds ratio so it is not necessary to include "The crude odds ratio ……..". Authors can add "Statistical significance was determined at a 95% confidence level" after "…………. "Between dependent and independent variables".	Thank you very much: This statement is modified per current analysis type.(**Line 38-4**2)
	• Line 39: Authors indicated in the methods section of the abstract that 809 respondents were used for the study. However, in the results section of the abstract, they indicated 729. The authors should clarify the inconsistency.	Thank you: 809 are all study units or what we planned But 729 are the only responded the questionnaires. The reason for the non-response participants were explained **(Line 386-391**)
	• Lines 41 – 42: Authors should indicate the unit of measurement of age and work experience.	Thank you. But the research team agreed to delete sociodemographic variable due less important for the readers in the abstract section

	• The results section is not very clear. Authors should consider re-writing the results to make them clearer.	Thank you it is rewrite it as you, other reviewers and research team agreed.
	Introduction<br>• Line 65: "They often cited" should be written as "They are often cited".	Thank you it is inserted (**Line 66**)
	• Lines 69 – 70: This needs to be revised to make it	Modified (**Line 68**)
	• Line 73: "but also they are facing with a variety of…" should be written as "but also they face several occupational ….".	Modified (**Line 74-84**)
	• Line 97: Reference (35) should be in square brackets, [35].	Thank you it was mistakenly cited by manually and it is correct in this version by ENDNOTE
	Methods<br>• Line 109 – 111: "In terms of study areas, public hospitals within a 200km radius of Harar were chosen from three regions and one administrative city". This statement is not	These statements are corrected base on what we done (**Line 113-117**)
	• Lines 114 – 117: "From ….." the content should be checked and it is a bit unclear.	Modified and detailed (**Line 117-120**)
	• Line 115: The word "daily" should be deleted.	Thank you it is deleted
	• Lines 116 – 117: The (mean+SD) values in the bracket are 4 but the parameters are three (bed occupancy, outpatient flow and inpatient). This should be rectified.	Thank you very much they separately stated in (**Line 121-122**)
	• Line 117: "Out of 5680 hospital staff of 5680" This statement should be checked as 5680 is repeated.	Thank you , it is revised [**Line 121-122**]
	• The map of the study area is blurred. Authors should replace it with a clear map.	It is modified with new one (**Line 124**)
	• Line 122: Section 2.2 "Source and study population" should be revised to read "Study population".	Thank you it is corrected (**Line 125)**
	• Line 134: The sentence is incomplete.	Thank you both inclusion and Exclusion criteria were revised and rewrote (**129-135**)
	• Line 135: Section 2.4 - Sample determination. The entire section is not clear. Authors should be specific on how they estimated the sample size for the study.	This sectional is modified well after team discussion (**Line 136-144**)
	• Line 151: Section 2.5 Selection Procedures should be written as "Sample selection procedures". Authors should explain how they arrived at the sample size for each of the four study States.	This sectional is also modified well after team discussion (**Line 144-149**)
	• Line 165: About Figure 2. According to the information in section 2.5, Dire Dawa City has a total sample size of 82. However, it says otherwise in the Figure. The same applies to the Oromia Regional state.	As justified under method section, two hospitals were randomly selected from four of the regions by providing equal change for each. Based on this Figure 2 is modified as the suggestions (**Line 144-149**)
	• Line 175: "………workers' representatives (KII-2) were purposely selected from each eight". The authors should check this statement again as there may be an error with the use of "eight".	Thank you. Corrected as from 8 hospitals, 8 IPC experts (KII-1) and 8 Sanitary representatives/Coordinators (KII-2) were used as KII(Line 136-138).. KII well explained in line **236-256.** Regarding their feedback **,s**ome of KII responses were changed into quantitative data in order to summarize rather explaining for each KII. Thus, average of their feedback was used and the key information was narrated for the readers (**Line 357-370**)
	• Lines 192 – 194: Authors should check the time shifts as they overlap and create confusion for readers.	It means job rotation for consecutive one month, now it is corrected

<table>
<tr><td></td><td>• Lines 228 – 246: The information presented here does not form part of quantitative data. Key informant interviews are ways of collecting qualitative data. Authors should kindly revise.</td><td>A lot of questions supported by closed and open for their opinion. Thus, both are included in narrative as well as quantitative based on adapted questionnaires</td></tr>
<tr><td></td><td>• Line 258: “independent variable” instead of "variable independent”</td><td>Thank you it is corrected (Line 181)</td></tr>
<tr><td></td><td>• Lines 267 – 274: Section 2.10 Ethical statement. Authors should take the entire section out.</td><td>Editor suggested “Ethical statement” in method section (Line 292)</td></tr>
<tr><td></td><td>Results
• Lines 288 – 289: “Of these, 43.59% (95%CI: 39.87, 47.42) and 50.00% (95%CI: 35.52, 64.47) were cleaners and solid waste collectors, respectively”. The percentages of cleaners and solid waste collectors should sum up to 100%. This needs to be cross-checked.</td><td>It was reported from this , but now statement is modified
<table>
<tr><td>Occupational injuries in the last 12 mont</td><td>Job categories
1. Cleane</td><td>
2. Waste</td><td>Total</td></tr>
<tr><td>0</td><td>383
56.41</td><td>25
50.00</td><td>408
55.97</td></tr>
<tr><td>1. Yes</td><td>296
43.59</td><td>25
50.00</td><td>321
44.03</td></tr>
<tr><td>Total</td><td>679
100.00</td><td>50
100.00</td><td>729
100.00</td></tr>
</table></td></tr>
<tr><td></td><td>• Table 3: Authors should provide a note under the table to explain the meaning of values with single asterisks.</td><td>We did it even in the current version under each table. Despite The previous Table 3 is removed due less significant in our work. We replaced it with Modified Poisson Regression Bivariate analysis of out put</td></tr>
<tr><td></td><td>• Line 330: Section 3.6 – Assessment from IPC as KII. This section per the methods is supposed to be qualitative. However, nothing shows under the results section. Authors should present their qualitative findings.</td><td>Thank you very much.KII recruited for both quantitative and some qualitative information. Therefore, almost the important issues regarding our findings are addressed in this version (Line 377-394)</td></tr>
<tr><td></td><td>Discussion
• The discussion could be improved by revising the qualitative aspect to reflect the suggested revision in the results section.</td><td>Thank you. The discussion and result section have been improved as editor and all of you suggested .</td></tr>
<tr><td></td><td>• Authors should only discuss the findings based on the objectives of the study and delete any other thing that is not related to the objectives of the study.</td><td>Thank you for the feedback. In this version, we in lighted to only the scope of study including qualitative evidence in order to rich the quantitative evidence</td></tr>
<tr><td></td><td>• The authors could improve the discussion by including the implications of the findings.</td><td>Now, as far as we concerned it is well illustrated in this version</td></tr>
<tr><td></td><td>Conclusion
• This will have to be reconsidered after effecting all the suggested corrections.</td><td>It is rewritten as suggestions</td></tr>
<tr><td></td><td>General comment
• There are many errors in the manuscript that need to be corrected. I suggest authors proofread the entire manuscript. Some parts have been highlighted yellow and green and I do not know the rationale for doing that. Authors should ensure they submit a clean document.</td><td>We would like to thank you once more for your amazing and intelligent contribution to our work, which has resulted in high-caliber paper to be published in PLOSONE</td></tr>
<tr><td></td><td>• A few mentions of the errors can be found in lines 53, 102, 103 115, 138 – 140, 192, 248 – 249, 256, 472, 473 – 474, and 486 – 487.</td><td>Thank you all these lines are improved. Despite of fact that due to reducing or adding additional information within the manuscript this lines are changed.</td></tr>
<tr><td></td><td>Reviewer #3:</td><td></td></tr>
<tr><td></td><td>Abstract:
In the methods, include the sampling technique and data collection methods. Include the statistical analysis in a</td><td>Thank you very much for kindly feedback. Abstract section is improved as you suggested considering the other feedback</td></tr>
</table>

	concise manner.<br>The results should include the factors found to increase the risk of occupational injuries. IPC should be provided in its full form.<br>Conclusions should be concise and based on important findings. Avoid making comments on policy implications that are not based on the findings.	
	Methods:<br>The purpose of mentioning mean, range, and standard deviation (mean ± SD) by bed occupancy (lines 114 to 117) is unclear.	The statements are modified based on suggestions. We interested to provide the information "how many the bed occupancy, outpatient flow and inpatients" in the current study settings as background.
	The sentence "Out of 5680… and waste collectors" (lines 117-118) is not clear	It is similar to above explanation,it was interested to give a number of staffs in the hospitals. Then we cascade the information to the study unit , in which we planned to study.
	In the inclusion criteria, it is mentioned that newly recruited SW working for more than a month were included in this study. This would bias the results because the occupational injuries that occurred within the last 12 months influence the findings of prevalence by the respondents who joined as SW for more than a month.	It is common the country , any civil service workers including sanitary workers those have less than a month work experience excluded because, they expected that they do not fully started their work at this stage, mean they are on training as well as some orientation about workplace
	Sample size determination: p-value for sample size calculation, p-value for age and PPE has been considered which is not appropriate, these should be avoided.	Thank you it is modified based on your and other reviewers considering mother document (main document we have) as based
	There is no sampling technique to include respondents<br>There should be criteria for selecting KIs (lines 175 to 177)<br>In independent variables (2.6.2), variable related to stress symptoms should be mentioned.	• Criteria was set (132-133)<br>• Job related symptom was defined **(line 223-225**)
	Results: The results indicate that 729 out of the 809 calculated samples were respondents. It's important to explain why others did not respond.<br>In Table 2, there is a mistake in the total number of categories in the columns..	Out of 809, about 729 sanitary workers were responded the questionnaires. About 80 of them were refused to participated. As majority of them reported to data collectors as: "We do not expect this study wouldn't give any guaranty or career development if we give an information (**Line 377-383**)
	Limitations:There may be a recall bias in reporting occupational injuries in the last 12 months; how this has been addressed should be mentioned	Thank you it is addressed **[Line 555-556**]
	Conclusions: The comments (lines 484–488) regarding policy implications are not supported by the findings; however, these can be discussed in discussion	Thank you, the conclusion section was modified and rewrote as suggestions [**Line 562-566**]
6.	Do you want your identity to be public for this peer review?<br>Reviewer #1: **Yes:** It is my review<br>Reviewer #2: No<br>Reviewer #3: No	Really, once again, thank you all you for your nice contribution on our paper

Regards,

Corresponding Author
