# Supplementary material for: Occupational injuries and associated factors among sanitary workers in public hospitals, eastern Ethiopia: A modified Poisson regression model analysis
Source: PLoS One. 2024 Nov 15;19(11):e0310970. doi: 10.1371/journal.pone.0310970 (PMC11567533; doi:10.1371/journal.pone.0310970)
Supplement: Attachment — Submitted filename: Response to Reviewers3.docx [file pone.0310970.s009.docx]

**June, 2024**

**Subject**: Sending revised manuscript, “**PONE-D-23-41265**”

**Greetings to**

- Reviewer #3

Kindly, as corresponding Author, on behalf of the rests, I would start by extending a warm greeting to the reviewer 3.Please allow me to express my gratitude to you for your sincere advice, criticisms, suggestions and recommendations. These contributions and efforts are very significant to the quality of our main manuscript in the current version.

Having this, we have used the following format of table to address all issues forward from you ,which embedded in the attached table. Within a manuscript, we used or highlighted with two colors: “Yellow”used for modification and “Cyan Green” used for newly added paragraphs addressed in “Revised Manuscript with Track Change”

Some comments on Knowledge , Attitude, Personal protective equipment and Infection prevention questions were raised from you and other . For the mentioned items, the questions were prepared as associated /determinants of occupational injuries, which are submitted as supplementary material. The response for each categorical variables designed to use for other, despite of fact that we used them as summary by giving cut point for each variable., which explained in the method section . Dear due to entire correction, some of line numbers are may move up or down. Thus, we expect to politely understanding this problems while you scan the comments or suggestion thought the manuscript.

| S.no | Reviewer #3 comments | Authors’ feedback |
| --- | --- | --- |
| 1. | Is the manuscript technically sound, and do the data support the conclusions? |  |
|  | Reviewer #3: Yes | Thank you very much for your encouragement |
| 2. | Has the statistical analysis been performed appropriately and rigorously? |  |
|  | Reviewer #3: Yes | Thank you very much for your encouragement |
| 3. | Have the authors made all data underlying the findings in their manuscript fully available? |  |
|  | Reviewer #3: Yes | Thank you very much for your encouragement |
| 4 | Is the manuscript presented in an intelligible fashion and written in standard English? |  |
|  | Reviewer #3: No | Thank you for your feedback, made this paper by your criticism |
| 5 | Comments by component |  |
|  | Abstract: In the methods, include the sampling technique and data collection methods. Include the statistical analysis in a concise manner. The results should include the factors found to increase the risk of occupational injuries. IPC should be provided in its full form. Conclusions should be concise and based on important findings. Avoid making comments on policy implications that are not based on the findings. | Thank you very much for kindly feedback. Abstract section is improved as you suggested considering the other feedback |
|  | Methods: The purpose of mentioning mean, range, and standard deviation (mean ± SD) by bed occupancy (lines 114 to 117) is unclear. | The statements are modified based on suggestions. We interested to provide the information “how many the bed occupancy, outpatient flow and inpatients” in the current study settings as background. |
|  | The sentence “Out of 5680… and waste collectors” (lines 117-118) is not clear | It is similar to above explanation,it was interested to give a number of staffs in the hospitals. Then we cascade the information to the study unit , in which we planned to study. |
|  | In the inclusion criteria, it is mentioned that newly recruited SW working for more than a month were included in this study. This would bias the results because the occupational injuries that occurred within the last 12 months influence the findings of prevalence by the respondents who joined as SW for more than a month. | It is common the country , any civil service workers including sanitary workers those have less than a month work experience excluded because, they expected that they do not fully started their work at this stage, mean they are on training as well as some orientation about workplace |
|  | Sample size determination: p-value for sample size calculation, p-value for age and PPE has been considered which is not appropriate, these should be avoided. | Thank you it is modified based on your and other reviewers considering mother document (main document we have) as based |
|  | There is no sampling technique to include respondents There should be criteria for selecting KIs (lines 175 to 177) In independent variables (2.6.2), variable related to stress symptoms should be mentioned. | - Criteria was set (132-133) - Job related symptom was defined **(line 223-225**) |
|  | Results: The results indicate that 729 out of the 809 calculated samples were respondents. It's important to explain why others did not respond. In Table 2, there is a mistake in the total number of categories in the columns.. | Out of 809, about 729 sanitary workers were responded the questionnaires. About 80 of them were refused to participated. As majority of them reported to data collectors as: “We do not expect this study wouldn’t give any guaranty or career development if we give an information (**Line 377-383**) |
|  | Limitations:There may be a recall bias in reporting occupational injuries in the last 12 months; how this has been addressed should be mentioned | Thank you it is addressed **[Line 555-556**] |
|  | Conclusions: The comments (lines 484–488) regarding policy implications are not supported by the findings; however, these can be discussed in discussion | Thank you, the conclusion section was modified and rewrote as suggestions [**Line 562-566**] |
| 6. | Do you want your identity to be public for this peer review?  Reviewer #3: No | Really, once again, thank you all you for your nice contribution on our paper |

Greetings, Dr./Prof.

Please come forward if you are not satisfied with our input so far on your comments or suggestion or recommendations ; we will take it from you and make the necessary corrections.

Sincerely

Corresponding Author
