# Supplementary material for: Occupational injuries and associated factors among sanitary workers in public hospitals, eastern Ethiopia: A modified Poisson regression model analysis
Source: PLoS One. 2024 Nov 15;19(11):e0310970. doi: 10.1371/journal.pone.0310970 (PMC11567533; doi:10.1371/journal.pone.0310970)
Supplement: Attachment — Submitted filename: Response to Reviewers1.docx [file pone.0310970.s010.docx]

**June, 2024**

**Subject**: Sending revised manuscript, “**PONE-D-23-41265**”

**Greetings to**

- Reviewer #1

Kindly, as corresponding Author, on behalf of the rests, I would start by extending a warm greeting to the reviewer 1. .Please allow me to express my gratitude to you for your sincere advice, criticisms, suggestions and recommendations. These contributions and efforts are very significant to the quality of our main manuscript in the current version.

Dear Having this, we have used the following format of table to address all issues forward from you ,which embedded in the attached table. Within a manuscript, we used or highlighted with two colors: “Yellow”used for modification in line to your comments and suggestions and also “Cyan Green” used for newly added paragraphs to rich the manuscript and keep the quality , both these addressed in “Revised Manuscript with Track Change”. We conceptualized the feedback within the table why we changed or modified based on your comments.

Kindly, some comments on Knowledge , Attitude, Personal protective equipment and Infection prevention questions were raised from you and other . For the mentioned items, the questions were prepared as associated /determinants of occupational injuries, which are submitted as supplementary material. The response for each categorical variables designed to use for other, despite of fact that we used them as summary by giving cut point for each variable., which explained in the method section. Dear due to entire correction, some of line numbers are may move up or down. Thus, we expect to politely understanding this problems while you scan the comments or suggestion thought the manuscript.

| S.no | Reviewer 1 comments | Authors’ feedback |
| --- | --- | --- |
| 1. | Is the manuscript technically sound, and do the data support the conclusions? |  |
|  | Reviewer #1: Yes | Thank you very much for your encouragement |
| 2. | Has the statistical analysis been performed appropriately and rigorously? |  |
|  | Reviewer #1: Yes | Thank you very much for your encouragement |
| 3. | Have the authors made all data underlying the findings in their manuscript fully available? |  |
|  | Reviewer #1: Yes | Thank you very much for your encouragement |
| 4 | Is the manuscript presented in an intelligible fashion and written in standard English? |  |
| 5 | Review by components |  |
|  | Reviewer #1: Yes | Thank you all for input |
|  | Abstract, line 33 delete from after sample size 809 | Thank you, it is corrected |
|  | Introduction Line 60, what do you mean collect solid garbage? Is there liquid garbage? | We corrected as “solid waste” |
|  | Method Line 117 what is mean Out of 5680 hospital staff of 568, about 809 of these were cleaners and waste collectors? What is your justification to take all sanitary workers as a sample? Why you no need sample among sanitary workers? | We used as background of the study. 5680 staffs are those working in selected public hospitals including sanitary workers. But, our plan was only for those staffs their works are associated with cleaning and waste disposing . These are accounted 809 . Despite the statement is improved |
|  | Line 135, If you took all sanitary workers, what is the need of sample size determination part?? Justify it? | This section is revised based mother document (Dissertation). Thus, the design effect =2 was added in this version |
|  | Line 121. The map of your study area needs scale, and proper naming of figure is required | The map is changed considering city where the study areas (hospitals) found |
|  | Line 151. 2.5 selection procedure. There is no proportional allocation, just you took the whole population? What is the need of stating sample selection? | This section was fully revised including entire figure 2 based on suggestion (**line 144-149**) |
|  | Line 153, they are not selected? |  |
|  | Line 172 your study period was from May 30th, 2023 to August 30th, 2023, why the title of figure 2 is 2022? | Thank you, its corrected “2023” |
|  | Line 175 unfinished sentence | Modified (**Line 224-226**) |
|  | Line 181. 2.6.2 Independent Variables, what is the difference between Institutional variables and Working environment variables? May be enabling variables? | We revised this section and added both together **(Line 207-214)** |
|  | Line 202 contents of the questionnaire not question? | Thank you, it modified as suggested (**line 198**) |
|  | Line 208 the 7 days Boolean logic questionnaire not mentioned in your abstract of line 34 | It is added per as suggestion (**line 39**) |
|  | Line 210, what are Determinants questions: eighteen standard questions were prepared for associated factors mean? Are they different from other independent variables? | In our work, we used Determinants or independent variables or associated factors exchangeable.. Now, it changed into “**Associated factor”** |
|  | Line 228 Key informant interview = Is it data collection or analysis part? Justify why you need to summarize like quantitative data? | Thus, we used some of them were for quantitative and s some of them qualitative. As you see some data converted into quantitative in order to compiled the data for easily understanding. |
|  | Line 247. What are the languages used for data collection? | Three languages: Somali, Oromo and Amharic, which are stated in data quality local language (**Line 263-264**) |
|  | Line 262, what is MLR? | Thank you:MLR meant that multiple logistic regression, but In this version it was changed into modified Poison regression. It has been changed based genuine comments from PLOS editor. Really, we were happy on it and because it is milestone for our work because the major weakness of logistic regression is that it tends to over-estimate the risk if the outcome of interest is not rare. However, modified Poisson regression model is preferred in cross-sectional studies when the outcome of interest is not rare because it approximates the risk ratios or relative ratios better than binary logistic regression model (**Line 292-296**) |
|  | Line 282 Table 1 Sociodemographic table: Is there separate division of labour for cleaners and waste collectors in the Ethiopian hospital human resource structure? Waste collectors from municipality or have their own? | Of course, yes. Waste collectors are recruited in some of hospitals due to large of solid waste produced in hospitals. |
|  | Line 288, Burden of occupational Injuries, the calculation and narration is not clear? | It was done as proportionally using their own denominator, that means 296/679 equals to 43.59% and 25/50 equals to 50.00% occupational injuries were cleaners and solid waste collectors, respectively.    By avoiding the above figures, it has been corrected as   1) 92.21% was for only cleaners in the hospitals 2) 7.79 % was for only waste collectors in the hospitals 3) 321/721 (44.03% ) was both of them    |
|  | Line 310, where do you get neutral attitude? | Attitude toward workplace risk was measured by likert scale (Assessment questions attached as supplementary material). The items then classified unfavored if socre <median(3.00) , neutral if score is 3.0 and favoured media is >3.00 **(Line 210-213**) |
|  | Table 3 and Table 4, If you ran logistic regression why you need to calculate X2 for comparison, please justify? | Table 3: Research team also agreed to delete Table 3 due to less significant for the readers |
|  | Table 4, Variables: Had MSD and conduct supervision are they significant? | According TO current analysis, those had MSD is contribution for likelihood of occupational Injuries at un-dajusted prevalence ratio(**Line 322**) while, lack of supervision had more likely to increase the risk of occupational injuries at adjusted prevalence ratio(**line 336**) |
|  | Line 321. How do you measure Knowledge, attitude and practice as shown in your Table 4? | Knowledge, attitude and practice were measured with standard tools. All cut point of other tools are illustrated (**Line 207-234**) |
|  | Line 330. 3.6 Assessment from IPC as KII, the analysis and result for Key Informant Interview is not clear please make it clear as qualitative data finding? | Thank you. KII well explained in line **236-256.** Regarding their feedback **,s**ome of KII responses were changed into quantitative data in order to summarize rather explaining for each KII. Thus, average of their feedback was used and the key information was narrated for the readers (**Line 357-370**). |
|  | Line 465, strength, the study had no sample, instead all populations assessed | Thank you very much it is corrected as suggestion **(Line 547**) |
|  | Line 480, Conclusion. You don’t have a variable indicating unsafe hygiene, how do you conclude in the first sentence? | Thank you: It modified **(Line 562-567**) |
|  | Line 484. Have you addressed the rules, policies and regulations related to OHS? | Thank you for your information. We removed from conclusion and added in the discussion with appropriate meaning (**Line 391-39**3). |
| 6 | Do you want your identity to be public for this peer review?  Reviewer #1: Yes , it is my review | Once again thank you the honesty and put your huge effort in our work |

Dear, Dr./Prof.

Please come forward if you are not satisfied with our input so far in your comments or suggestions ; we will take it from you and make the necessary corrections.

Sincerely

Corresponding Author
