# Supplementary material for: Occupational injuries and associated factors among sanitary workers in public hospitals, eastern Ethiopia: A modified Poisson regression model analysis
Source: PLoS One. 2024 Nov 15;19(11):e0310970. doi: 10.1371/journal.pone.0310970 (PMC11567533; doi:10.1371/journal.pone.0310970)
Supplement: Attachment — Submitted filename: Response to Reviewers3_F.docx [file pone.0310970.s011.docx]

**September, 2024**

**Subject**: Sending revised manuscript, “**PONE-D-23-41265**”

**Kindly to**

- Reviewer #3

Kindly, as corresponding Author, on behalf of the rests, I would start by extending a warm greeting to the reviewer 3 .Please allow me to express my gratitude to you for your sincere advice, criticisms, suggestions and recommendations. These contributions and efforts are very significant for the quality of our current paper.

Dear Having this, we have used the following format of table to address all issues forward from you ,which embedded in the attached table. Within a manuscript, we used or highlighted with two colors: “Yellow”used for modification in line to your comments and suggestions and also “Cyan Green” used for newly added paragraphs to rich the manuscript and keep the quality , both these addressed in “Revised Manuscript with Track Change”. We conceptualized the feedback within the table why we changed or modified based on your comments.

Kindly, Dear due to entire correction, some of line numbers are may move up or down. Thus, we expect to politely understanding this problems while you scan the comments or suggestion thought the manuscript.

| S.no | Reviewer 3 comments | Authors’ feedback |
| --- | --- | --- |
| 1. | If the authors have adequately addressed your comments raised in a previous round of review and you feel that this manuscript is now acceptable for publication, you may indicate that here to bypass the “Comments to the Author” section, enter your conflict of interest statement in the “Confidential to Editor” section, and submit your "Accept" recommendation. **Reviewer #3: Yes** | Thank you very much for your overall your contribution in our current status of the paper’s quality |
| 2. | Is the manuscript technically sound, and do the data support the conclusions? The manuscript must describe a technically sound piece of scientific research with data that supports the conclusions. Experiments must have been conducted rigorously, with appropriate controls, replication, and sample sizes. The conclusions must be drawn appropriately based on the data presented. **Reviewer #3: Yes** | Thank you very much for your encouragement |
| 3. | Has the statistical analysis been performed appropriately and rigorously? **Reviewer #3: Yes** | Thank you very much for your encouragement |
| 4 | Have the authors made all data underlying the findings in their manuscript fully available? The PLOS Data policy requires authors to make all data underlying the findings described in their manuscript fully available without restriction, with rare exception (please refer to the Data Availability Statement in the manuscript PDF file). The data should be provided as part of the manuscript or its supporting information, or deposited to a public repository. For example, in addition to summary statistics, the data points behind means, medians and variance measures should be available. If there are restrictions on publicly sharing data—e.g. participant privacy or use of data from a third party—those must be specified. **Reviewer #3: Yes** | Thank you very much for your encouragement |
| 5 | Is the manuscript presented in an intelligible fashion and written in standard English? PLOS ONE does not copyedit accepted manuscripts, so the language in submitted articles must be clear, correct, and unambiguous. Any typographical or grammatical errors should be corrected at revision, so please note any specific errors here. **Reviewer #3: Yes** | Once again, than you very much |
| 6 | Review Comments to the Author Please use the space provided to explain your answers to the questions above. You may also include additional comments for the author, including concerns about dual publication, research ethics, or publication ethics. (Please upload your review as an attachment if it exceeds 20,000 characters) **Reviewer comments #3 Abstract** |  |
|  | - Thank you, authors; you have addressed all my queries and comments satisfactorily.However, Table 2 is too long; for better understanding, separate it into 'injury type', 'body part injured', and 'reasons for injuries'. There is still scope for editing to further improve the English language. | - Thank you , three of them were modified as suggestion, but due to interest to show the number of case, we use as **'injury type**(n=321) |
|  | - There is still scope for editing to further improve the English language. | - Thank you, all parts from Abstarct to end of coclusion have been checked, modified and well rewritten based on your comments, reviewer 1 and reviewer 2 |
| 7 | - PLOS authors have the option to publish the peer review history of their article (what does this mean?). If published, this will include your full peer review and any attached files. If you choose “no”, your identity will remain anonymous but your review may still be made public. Do you want your identity to be public for this peer review? **Reviewer #3: No** | - Once again thank you very much for huge contribution on our work. |

Dear Prof/Dr. Reviewer 3, Once again thank you very much for incredible comments, critism and suggestion for our paper we have now. We learn a lot of skill and got a lot of experience from you

Sincerely

Corresponding Author
