# Supplementary material for: Occupational injuries and associated factors among sanitary workers in public hospitals, eastern Ethiopia: A modified Poisson regression model analysis
Source: PLoS One. 2024 Nov 15;19(11):e0310970. doi: 10.1371/journal.pone.0310970 (PMC11567533; doi:10.1371/journal.pone.0310970)
Supplement: Attachment — Submitted filename: Reviewer comments.docx [file pone.0310970.s006.docx]

Reviewer comments

Manuscript Number = PONE-D-23-41265

Title: Burden and determinants of occupational injuries among sanitary workers in public hospitals, eastern Ethiopia

Dear authors, you need to address and clarify the following comments before publication

**Abstract**, line 33 delete from after sample size 809

**Introduction**

Line 60, what do you mean collect solid garbage? Is there liquid garbage?

**Method**

Line 117 what is mean Out of 5680 hospital staff of 568, about 809 of these were cleaners and waste collectors? What is your justification to take all sanitary workers as a sample? Why you no need sample among sanitary workers?

Line 135, If you took all sanitary workers, what is the need of sample size determination part?? Justify it?

Line 121. The map of your study area needs scale, and proper naming of figure is required

Line 151. 2.5 selection procedure. There is no proportional allocation, just you took the whole population? What is the need of stating sample selection?

Line 153, they are not selected?

Line 172 your study period was from May 30th, 2023 to August 30th, 2023, why the title of figure 2 is 2022?

Line 175 unfinished sentence

Line 181. 2.6.2 Independent Variables, what is the difference between Institutional variables and Working environment variables? May be enabling variables?

Line 202 contents of the questionnaire not question?

Line 208 the 7 days Boolean logic questionnaire not mentioned in your abstract of line 34

Line 210, what are Determinants questions: eighteen standard questions were prepared for associated factors mean? Are they different from other independent variables?

Line 228 Key informant interview = Is it data collection or analysis part? Justify why you need to summarize like quantitative data?

Line 247. What are the languages used for data collection?

Line 262, what is MRL?

Line 282 Table 1 Sociodemographic table: Is there separate division of labour for cleaners and waste collectors in the Ethiopian hospital human resource structure? Waste collectors from municipality or have their own?

Line 288, Burden of occupational Injuries, the calculation and narration is not clear?

Line 310, where do you get neutral attitude?

Table 3 and Table 4, If you ran logistic regression why you need to calculate X^2^ for comparison, please justify?

Table 4, Variables, Sex, Had MSD and conduct supervision are they significant?

Line 321. How do you measure Knowledge, attitude and practice as shown in your Table 4?

Line 330. 3.6 Assessment from IPC as KII, the analysis and result for Key Informant Interview is not clear please make it clear as qualitative data finding?

Line 465, strength, the study had no sample, instead all populations assessed

Line 480, Conclusion. You don’t have a variable indicating unsafe hygiene, how do you conclude in the first sentence?

Line 484. Have you addressed the rules , policies and regulations related to OHS?

Thank you
